# Dissecting the single-cell transcriptome network in patients with esophageal squamous cell carcinoma receiving operative paclitaxel plus platinum chemotherapy

**DOI:** 10.1038/s41389-021-00359-2

**Published:** 2021-10-26

**Authors:** Zhencong Chen, Yiwei Huang, Zhengyang Hu, Mengnan Zhao, Yunyi Bian, Zongwei Chen, Yuansheng Zheng, Guoshu Bi, Yanrui Pang, Cheng Zhan, Zongwu Lin, Weigang Guo, Qun Wang, Lijie Tan

**Affiliations:** 1 Department of Thoracic Surgery, Zhongshan Hospital, Fudan University, No. 180, Fenglin Road, 200032 Shanghai, China; 2 Department of Pathology, Zhongshan Hospital, Fudan University, No. 180, Fenglin Road, 200032 Shanghai, China

**Keywords:** Cancer, Immunology

## Abstract

Esophageal squamous cell carcinoma (ESCC) accounts for 90% of all cases of esophageal cancers worldwide. Although neoadjuvant chemotherapy (NACT-ESCC) improves the survival of ESCC patients, the five-year survival rate of these patients is dismal. The tumor microenvironment (TME) and tumor heterogeneity decrease the efficacy of ESCC therapy. In our study, 113,581 cells obtained from five ESCC patients who underwent surgery alone (SA-ESCC) and five patients who underwent preoperative paclitaxel plus platinum chemotherapy (NACT-ESCC), were used for scRNA-seq analysis to explore molecular and cellular reprogramming patterns. The results showed samples from NACT-ESCC patients exhibited the characteristics of malignant cells and TME unlike samples from SA-ESCC patients. Cancer cells from NACT-ESCC samples were mainly at the ‘intermediate transient stage’. Stromal cell dynamics showed molecular and functional shifts that formed the immune-activation microenvironment. APOE, APOC1, and SPP1 were highly expressed in tumor-associated macrophages resulting in anti-inflammatory macrophage phenotypes. Levels of CD8+ T cells between SA-ESCC and NACT-ESCC tissues were significantly different. Immune checkpoints analysis revealed that LAG3 is a potential immunotherapeutic target for both NACT-ESCC and SA-ESCC patients. Cell–cell interactions analysis showed the complex cell-cell communication networks in the TME. In summary, our findings elucidate on the molecular and cellular reprogramming of NACT-ESCC and ESCC patients. These findings provide information on the potential diagnostic and therapeutic targets for ESCC patients.

## Introduction

Globally, esophageal cancer is the sixth leading cause of cancer-related mortalities. In 2018, approximately 572,000 people were diagnosed with esophageal cancer while 509,000 esophageal cancer-related mortalities were reported worldwide [1]. Esophageal squamous cell carcinoma (ESCC) and esophageal adenocarcinoma (EA) are the most common histologic subtypes of esophageal cancer. In low-income countries, ESCC accounts for over 90% of all esophageal cancer cases [2–4]. In recent years, preoperative chemotherapy, also referred to as neoadjuvant chemotherapy (NACT), has significantly improved the survival outcomes of ESCC patients [5, 6]. However, Leng et al. [7] reported that approximately 13.9% of ESCC patients have a recurrence and distant organ metastasis after receiving a regimen of NACT combined with surgery, therefore, the prognosis of ESCC patients is poor [8–10]. There is a need to explore molecular and cellular reprogramming under SA-ESCC and NACT-ESCC conditions in order to identify potential therapeutic and prognostic targets.

Advances in single-cell technologies, especially single-cell RNA sequencing (scRNA-seq), have provided a new approach in profiling individual tumor cells, determining their roles, and exploring complex biological systems [11]. In this study, we used scRNA-seq to investigate the characteristics of individual cells in SA-ESCC and NACT-ESCC samples and explore the effects of neoadjuvant treatments on survival outcomes of ESCC patients. In summary, we investigated the outcomes of preoperative paclitaxel plus platinum chemotherapy on TME, immune cell infiltration, and overall survival for esophageal cancer.

## Results

### Global single-cell transcriptomic profiling of NACT-ESCC and ESCC

**Fig. 1:**
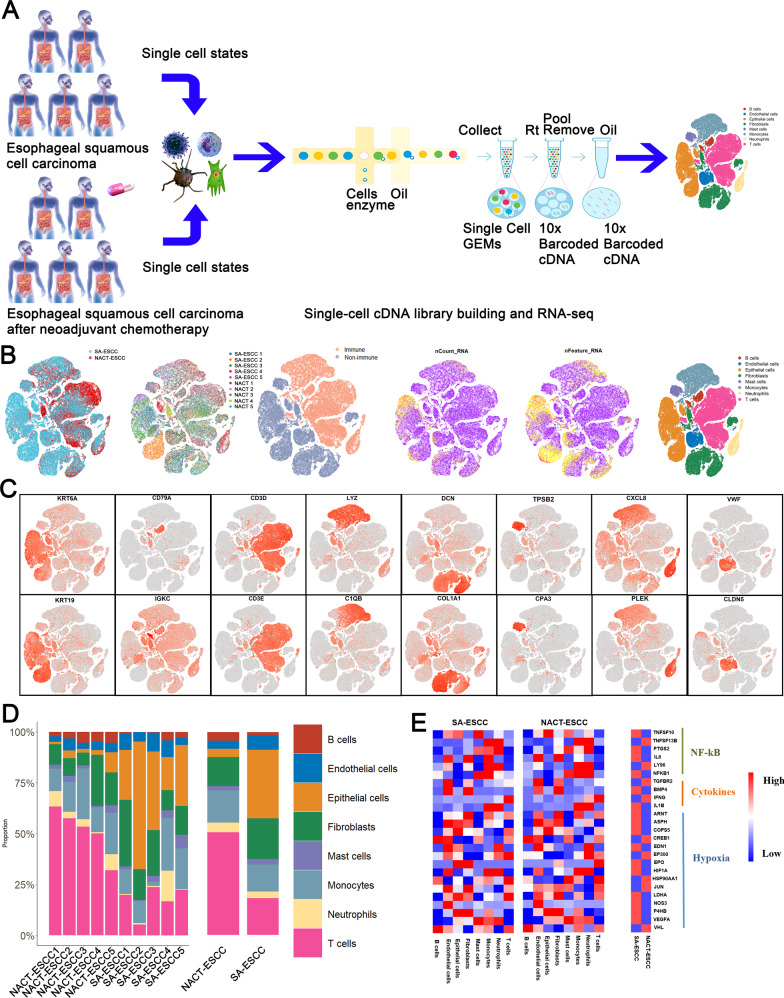
A Single-Cell Atlas of SA-ESCC and NACT-ESCC. **A** The workflow showing the collection and processing of specimens from SA-ESCC and NACT-ESCC tissues for scRNA-seq analysis. **B** TSNE of 113,581 cells, each cell has a color code. From left to right are: origin of sample type (SA-ESCC or NACT-ESCC), the corresponding patient, immune type, transcript counts, transcript features, and associated cell type. **C** Expression of marker genes for each cell subtype. **D** The proportion of each cell type in SA-ESCC and NACT-ESCC samples. **E** Heatmap of representative genes in cytokines, nuclear factor-kB (NF-kB), and hypoxia signaling pathways mapped onto cell types in SA-ESCC and NACT-ESCC samples.

Five SA-ESCC and five NACT-ESCC patients (Methods detailed) from FDZSH were enrolled in this study to investigate the cellular landscape of SA-ESCC and NACT-ESCC (Fig. 1A and Supplementary Table 1). After quality control, a total of 113,581 cells (including 63,837 SA-ESCC derived cells and 49,744 NACT-ESCC derived cells) were selected for subsequent analyses. Based on SingleR package, CellMarker dataset, and previous studies, cells were classified as epithelial, stromal (fibroblasts and endothelial cells), and immune cells (T, NK, B, Myeloid, and Mast cells) (Fig. 1B, C).

Figure 1D and Supplementary Fig. 1A show that NACT-ESCC patients had a high proportion of immune cells (especially T and B cells), while the proportions of epithelial and stromal cells were significantly low in NACT-ESCC patients, when compared to SA-ESCC patients. Additionally, our flow cytometry analysis revealed that compared to SA-ESCC, after being administered with preoperative chemotherapy, there was an obvious increase in immune cell numbers and a reduction in epithelial and stromal cells (Supplementary Fig. 1B). These findings imply that host anti-tumor immune responses were boosted by NACT while cellular compositions indicate gross alterations in SA-ESCC and NACT-ESCC patients, implying that adaptive immune responses were activated in these patients.

Cytokines, nuclear factor-kB (NF-kB), and hypoxia signaling pathways play an essential role in tumorigenesis [12, 13]. Therefore, we plotted cellular origins for some of the mediators of these pathways in our study (Fig. 1E). The expression levels of cytokines and NF-kB related genes (e.g., TNFSF13B, IL6, and NKFB1) were elevated in monocytes, implying that monocytes play a key role in promoting esophageal tumorigenesis. Moreover, compared to NACT-ESCC, hypoxia genes (e.g., LDHA, HIF1A, and EPO) were highly expressed in SA-ESCC patients, implying that chemotherapy affects the hypoxic microenvironment. Intriguingly, an increase in T/B cells was observed in NACT-ESCC samples, while the cytokines determined were not correlated with T/B cell functions, indicating that the increase in the number of T/B cells does not represent enhanced T/B cell functions. More research would be needed to address these concerns.

### Intrinsic tumor signatures associated with NACT-ESCC

**Fig. 2:**
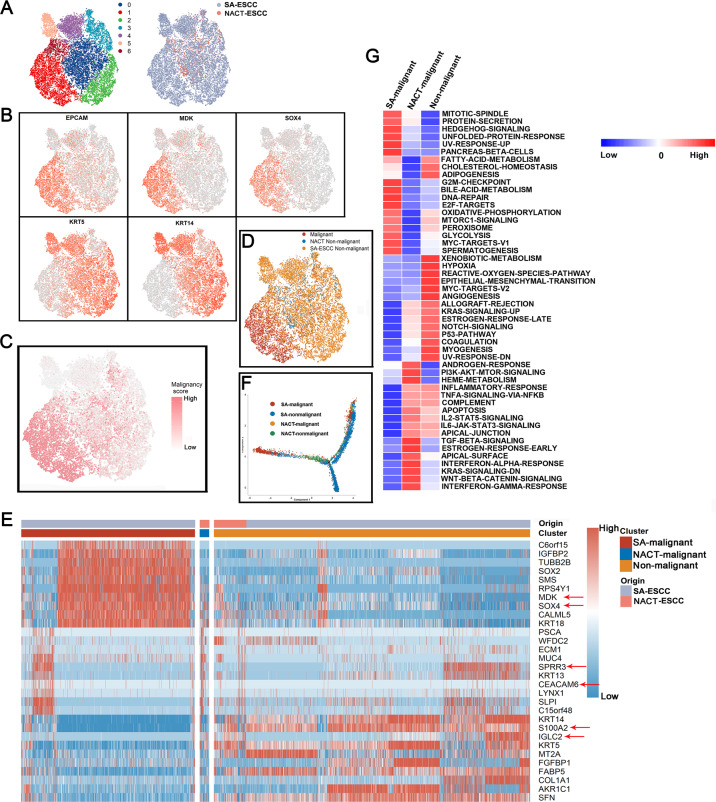
The single-cell transcriptomes of epithelial cells in non-malignant and malignant esophagus. **A** The TSNE plot and overview of the 90,606 epithelial cells, each cell has color code for its cluster and origin of sample type. **B** Expression of marker genes for each epithelial subtype. **C** The TSNE plot of epithelial cells colored based on the malignancy scores of the cells. **D** The TSNE plot of epithelial cells colored based on the cell type. **E** Expression of marker genes for SA-malignant, NACT-malignant, and Non-malignant cells. **F** Unsupervised transcriptional trajectory of malignant and normal epithelial cells from Monocle2 colored by cell type. **G** Differences in pathway activities scored per cell by GSVA among different epithelial cells subtypes. Normalized pathways scores.

After re-clustering epithelial cells, 7 clusters were identified (Fig. 2A). Copy number variations (CNVs) and marker genes were used to accurately separate tumor and non-malignant epithelial cells in SA-ESCC and NACT-ESCC samples. Tumor epithelial cell markers (EPCAM, MDK, and SOX4) were highly expressed in clusters 1 and 6 while non-malignant epithelial cell markers (KRT5 and KRT14) were highly expressed in clusters 0, 2, 4, and 5. Tumor and healthy epithelial cell markers were expressed in cluster 3 (Fig. 2B). These results indicate that clusters with high expression levels of healthy epithelial cell markers are the ‘normal epithelial cells, whereas clusters with high expression levels of tumor epithelial cell markers are ‘tumor epithelial cells. Genetic aberrations by inferring copy number variations (CNVs) revealed that ‘tumor epithelial cells clusters’ exhibited significantly higher malignant scores when compared to ‘normal epithelial cell clusters’ (Fig. 2C).

The R package ‘scpred’ was used to confirm malignant and non-malignant epithelial cells. It was found that 86% (3662/4245) of malignant epithelial cells were not identified as healthy epithelial cells in ‘scPred object’. In addition, 95% (6977/7324) of non-malignant epithelial cells were not identified as tumor epithelial cells in scPred object (Supplementary Fig. 1C). There was a high accuracy in cell annotation (Fig. 2D).

Differential expression analysis was performed to identify epithelial cell marker genes. Genes associated with poor prognosis (e.g., SOX4 and MDK) were mainly expressed in SA-ESCC-malignant epithelial cells. Besides, immune-associated genes (e.g., IGLC2, FABP5, and S100A2) were found to be upregulated in non-malignant epithelial cells. Notably, SPRR3 and CEACAM6 were found to be marker genes for NACT-malignant epithelial cells (Fig. 2E). Intriguingly, we found that non-malignant clusters could be divided into two sub-clusters based on their origin, implying the heterogeneity of non-malignant epithelial cells.

Flow cytometry and qRT-PCR were used to validate the differentially expressed genes in single-cell analysis. We focused on EPCAM+ and KRT5+ cells in NACT-ESCC and SA-ESCC patients (Supplementary Fig. 2A). Notably, qRT-PCR results revealed the differentially expressed genes in different epithelial cells (Supplementary Fig. 2B). Expression levels of SOX4 and MDK were significantly elevated in SA-ESCC-malignant epithelial cells compared to non-malignant epithelial cells (*P* < 0.01). Moreover, WFDC2 and MUC4 expression levels were significantly elevated in NACT-ESCC epithelial cells (*P* < 0.01). In contrast, expression levels of KRT14 and S100A2 were significantly elevated in non-malignant epithelial cells, when compared to malignant epithelial cells (*P* < 0.01). These findings are consistent with those of differential expression analysis in our study. Furthermore, expression levels of NACT-malignant epithelial cell marker genes were elevated in partial SA-ESCC-malignant and non-malignant epithelial cell clusters, implying that malignant epithelial cells in NACT-ESCC might be ‘intermediate transient cells’ between malignant cells in SA-ESCC and non-malignant epithelial cells.

Trajectory analysis was performed to quantitatively track the reprogramming of epithelial cells in SA-ESCC patients and NACT-ESCC patients (“Methods” detailed). Figure 2F shows that the differentiation trajectory of epithelial cells began with non-malignant epithelial cells derived from SA-ESCC patients, which later transitioned to NACT-derived non-malignant epithelial cells. Subsequently, epithelial cells were bifurcated into the SA-ESCC-malignant epithelial cell cluster or into the cluster with epithelial cells from different sources (NACT-ESCC or SA-ESCC patients). These results reveal the complex reprogramming of epithelial cells in SA-ESCC and NACT-ESCC.

The roles of different epithelial cell clusters were further characterized by comparing pathway activities (Fig. 2G). Comparisons of non-malignant and malignant epithelial cells revealed that TME formation related pathways (e.g., hypoxia and angiogenesis) were mainly upregulated in non-malignant epithelial cells. In addition, pathways associated with ESCC development and progression (e.g., G2M-checkpoint and DNA-repair pathways) were relatively upregulated in SA-ESCC malignant epithelial cells, when compared to non-malignant cells. Moreover, NACT-ESCC malignant epithelial cells and non-malignant epithelial cells exhibited some commonly activated pathways e.g., inflammatory response, and IL2/IL6 related signaling pathways.

### Construction of the single-cell network in SA-ESCC and NACT-ESCC

**Fig. 3:**
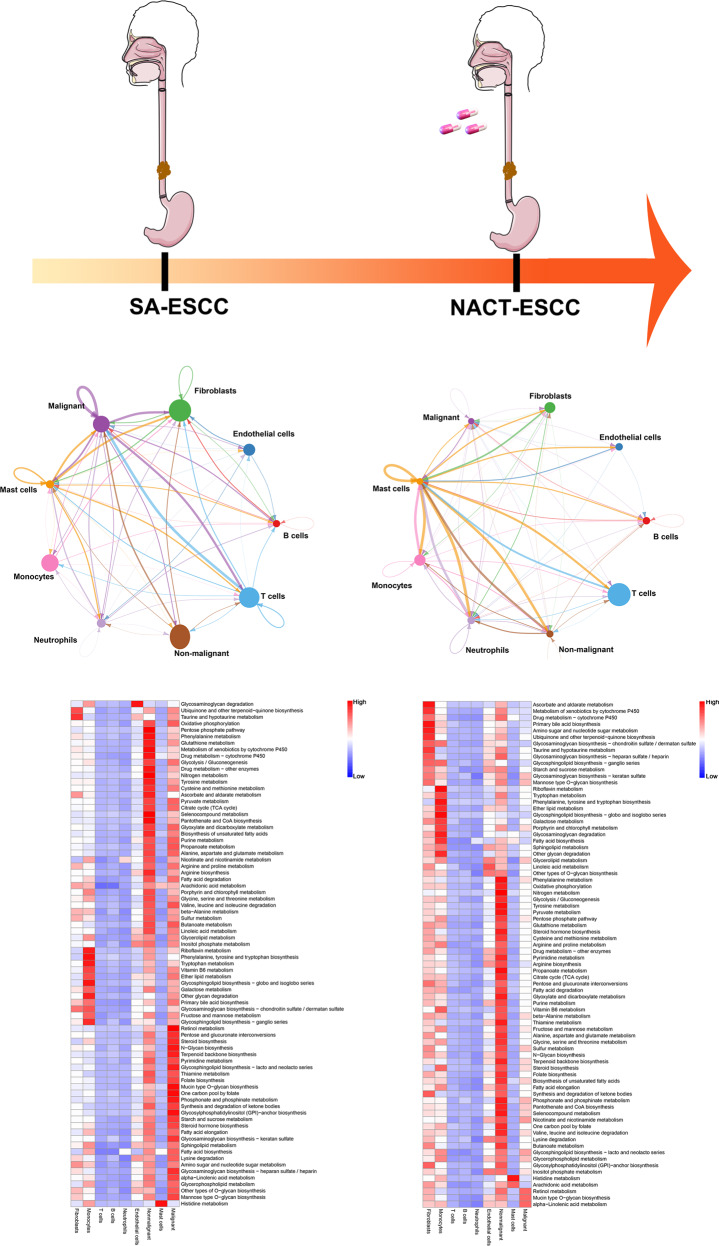
The single-cell transcriptome network underlying SA-ESCC and NACT-ESCC conditions. Cellular and molecular changes from SA-ESCC to NACT-ESCC. Top: The cell-cell communication networks constructed using CellPhoneDB. The nodes stand for each epithelial cell type in SA-ESCC and NACT-ESCC tissue, and the thickness of edges in the network denotes the correlation coefficient between each cell type. Bottom: Epithelial cell type-specific metabolic reprogramming based on scRNA-seq data under SA-ESCC and NACT-ESCC patients.

A single-cell transcriptomic network was established by describing correlations between each pair of cell populations to comprehensively evaluate cellular and molecular changes from SA-ESCC to NACT-ESCC (“Methods” detailed). Stromal cells in the SA-ESCC group, especially fibroblasts, highly communicated with other cell types (Fig. 3). In contrast, immune cells, especially T cells and monocytes in NACT-ESCC patients were found to play a major role in TME. Then, functional enrichment analyses, based on marker genes (top 50) for immune cells in NACT-ESCC and marker genes of stromal cells in SA-ESCC patients were performed (“Methods” detailed). It was found that stromal cells in SA-ESCC conditions were enriched in regulating extracellular matrix, whereas myeloid and T cells were implicated in the inhibition of tumor progression in NACT-ESCC patients (Supplementary Fig. 3). These results imply that chemotherapy alters the TME of SA-ESCC and activates the immune system to destroy tumor cells in NACT-ESCC.

Next, metabolic analysis was performed to investigate metabolic differences between the SA-ESCC and NACT-ESCC groups. Significantly upregulated metabolic pathways were found to be enriched in malignant and non-malignant epithelial cells of SA-ESCC (Fig. 3). The number of metabolic pathways in malignant epithelial cells were significantly low, and significantly upregulated in stromal and monocytes in NACT-ESCC. Oxidative phosphorylation, glycolysis/gluconeogenesis, and citrate cycle (TCA cycle) pathways, which are associated with mitochondrial activity, were found to be upregulated in both malignant and non-malignant epithelial cells in SA-ESCC. In the NACT-ESCC group, these pathways were only upregulated in non-malignant epithelial cells. Therefore, NACT-ESCC has a unique TME compared to SA-ESCC, and the mitochondria is a potential therapeutic target for SA-ESCC.

### Myofibroblast and tumor endothelial cell levels were significantly suppressed after chemotherapeutic administration

**Fig. 4:**
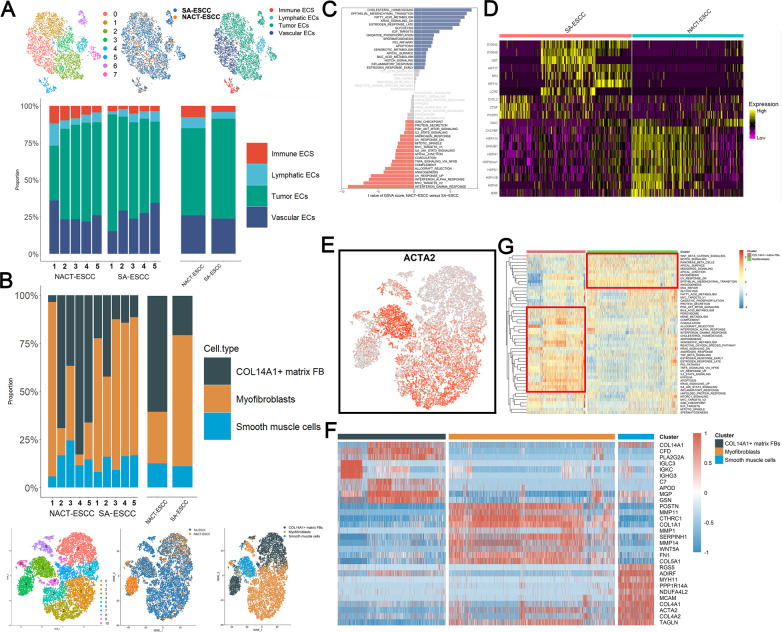
The scRNA Profiles for Stromal Cell Lineages in SA-ESCC and NACT-ESCC. **A** The TSNE plot and the proportion of endothelial cells in SA-ESC and NACT-ESCC samples. **B** The TSNE plot and proportion of fibroblasts in SA-ESC and NACT-ESCC samples. **C** Differences in pathway activities scored per cell by GSVA among immune EDCs under SA-ESCC and NACT-ESCC conditions. Normalized pathways scores. **D** Heatmap showing the activity of marker genes of immune EDCs in SA-ESCC and NACT-ESCC conditions. **E** Feature plot of ACTA2 in fibroblasts. **F** Heatmap showing the activity of marker genes in each fibroblasts subtypes. **(G)** Differences in pathway activities scored per cell by GSVA among COL14A1+ matrix fibroblasts and myofibroblasts. Normalized pathways scores.

A total of 19,977 and 6588 fibroblasts and endothelial cells (EDCs) were selected and re-clustered to characterize stromal cell dynamics in the tumor microenvironment. The expressions levels of CLDN5 and RAMP2 were used to identify endothelial cells. Sub-clustering of endothelial cells resulted in eight clusters (Fig. 4A). Endothelial cells from different sources were mixed and assigned to four cell types, including immune EDCs (CCL5 and CXCL13), lymphatic EDCs (PDPN and PROX1), tumor EDCs (ACKR1 and POSTN), and vascular EDCs (PLVAP and SLC9A3R2). Fibroblasts were confirmed by expressions of C1R and COL1A2, and 19,977 cells were selected for re-clustering analysis. A total of 11 sub-clusters were identified and assigned to three known cell types, including COL14A1 matrix fibroblasts (COL14A1 and GSN), myofibroblasts (CTHRC1 and MMP11), and vascular smooth muscle cells (DES and MYH11) (Fig. 4B). Details regarding marker genes of each cell type are shown in Supplementary Fig. 4A, B.

The proportion of tumor EDCs was low, while levels of immune EDCs were elevated in NACT-ESCC compared to EDCs in the SA-ESCC group (Fig. 4A). Therefore, tumor and immune EDCs were used for downstream analyses. GSVA analysis revealed that metabolic pathways, including cholesterol homeostasis and fatty acid metabolism, were activated in tumor EDCs in NACT-ESCC (Fig. 4C). In contrast, tumor EDCs from ESCC were found to be enriched in pathways that regulate endothelial cell development and cell fate (e.g., MYC related pathways and angiogenesis) [14, 15]. Analysis of differentially expressed genes between immune EDCs from SA-ESCC and NACT-ESCC revealed that cancer-promoting genes (e.g., S100A family genes) were highly expressed in SA-ESCC [16, 17] (Fig. 4D), while expressions of HSP family genes were significantly elevated in the NACT-ESCC group. Overexpressed HSP family genes have been reported to promote cancer growth and metastasis in several tumors, implying that HSP family genes are potential therapeutic targets for NACT-ESCC [18, 19]. In addition, functional enrichment analyses revealed that marker genes for SA-ESCC derived immune EDCs were implicated in biological regulation and cell growth, whereas marker genes for immune EDCs in the NACT-ESCC group were mainly involved in cellular and immune responses (Supplementary Fig. 4C).

Myofibroblasts play a key role in tumor progression, and have been described as cancer-associated fibroblasts, whereas COL14A1+ matrix fibroblasts have been reported in the non-malignant stromal environment [20, 21]. We found that COL14A1+ matrix fibroblasts were mainly enriched in NACT-ESCC, compared to SA-ESCC, whereas myofibroblasts were mainly enriched in the SA-ESCC group (Fig. 4B). Alpha smooth muscle actin (α-SMA) (ACTA2 gene product), was used as the marker protein for myofibroblasts to confirm their infiltration. ACTA2 was found to be mainly expressed in myofibroblasts and smooth muscle cells, which explains their contractile ability (Fig. 4E). In addition, POSTN and WNT5A, which are closely correlated with Wnt and Notch signaling pathways [22], were found to be highly expressed in myofibroblasts (Fig. 4F). Moreover, GSVA analysis revealed that the Wnt and Notch signaling pathways were enriched in myofibroblasts. COL14A1 + matrix fibroblasts play a vital role in defenses against abnormal stress levels in the stromal microenvironment (Fig. 4G).

### Macrophages play different roles in NACT-ESCC and SA-ESCC

**Fig. 5:**
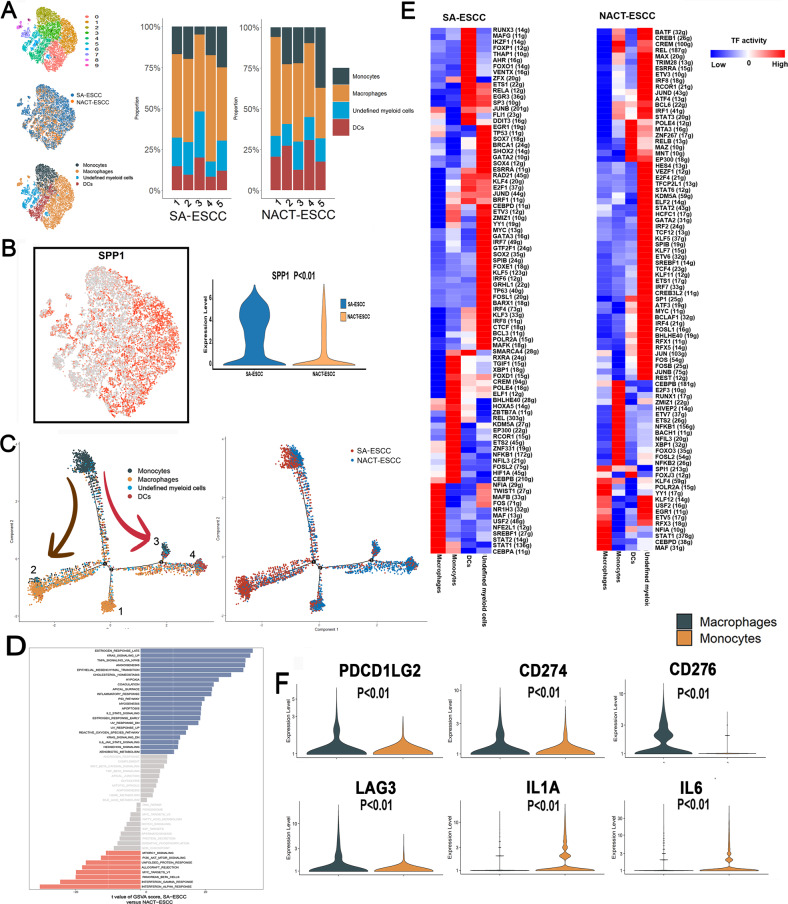
The scRNA Profiles for myeloid cells in SA-ESCC and NACT-ESCC. **A** The TSNE plot and the proportion of myeloid cells in SA-ESC and NACT-ESCC samples. **B** Feature plot and violin plot of SPP1. **C** Potential developmental trajectory of myeloid cells inferred by analysis with Monocle2. **D** Differences in pathway activities scored per cell by GSVA among macrophages in SA-ESCC and NACT-ESCC conditions. Normalized pathways scores. **E** Heatmap showing the activity of TFs in each myeloid cell subtypes in NACT-ESCC and SA-ESCC, respectively. The TF activity is scored using AUCell. **F** Violin plots of immune checkpoints upregulated or downregulated between monocytes and macrophages cells.

Based on the expressions of CD68 and LYZ, a total of 16,305 myeloid cells were selected and re-clustered, resulting in ten clusters, mainly comprising monocytes, macrophages, dendritic cells, and undefined myeloid cells (Fig. 5A and Supplementary Fig. 5A). The NACT-ESCC group exhibited reduced macrophage levels and elevated monocyte levels when compared to the SA-ESCC group (Fig. 5A). In addition, endo calcium-binding proteins, including S100A8, S100A9, and S100A12, which promote inflammatory responses were highly expressed in monocytes; whereas APOE and APOC1, which are associated with an anti-inflammatory phenotype [23, 24], were highly expressed in macrophages (Supplementary Fig. 5B). These findings imply that macrophages play different roles in NACT-ESCC and SA-ESCC.

Secreted phosphoprotein1 (SPP1), which suppresses immune responses associated with osteopontin, is a poor prognostic marker for several tumors [25, 26]. Mapping of the expression of SPP1 in myeloid sub-cell types revealed that SPP1 (Fig. 5B) is mainly expressed in macrophages, with higher levels in macrophages derived from SA-ESCC. Additionally, our immunofluorescence also confirmed that SPP1 was significantly downregulated in NACT-ESCC (Supplementary Fig. 6A). Trajectory analysis was performed to elucidate on transcriptional transition from monocytes to macrophages. State 1 and 2 consisted of macrophages from NACT-ESCC and SA-ESCC, respectively, while states 3 and 4 exhibited diverse functional cells that mainly originated from NACT-ESCC (Fig. 5C). In addition, GSVA analysis of macrophages revealed that pathways associated with anti-tumor (e.g., Interferon-gamma (IFN-gamma) and Interferon-alpha (IFN-alpha)) response pathways were enriched in NACT-ESCC. Pathways implicated in pro-tumor responses (e.g., KRAS and EMT pathways) were enriched in macrophages from SA-ESCC (Fig. 5D). The TNF-α-NF-κB pathway was also enriched in macrophages from SA-ESCC, implying that SA-ESCC macrophages in the TME enhance tumor development and dissemination, consistent with previous studies [27, 28].

SCENIC analysis was performed on NACT-ESCC and SA-ESCC cells (Fig. 5E and Supplementary Fig. 5C). In SA-ESCC, NFIA, TWIST1, and MAFB were identified as top 3 candidate TFs responsible for gene expression differences in macrophages. RXRA, TGIF1, and XBP1 were identified as the top 3 upregulated candidate TFs in monocytes from SA-ESCC conditions (Fig. 5E). Besides, expression levels of genes regulated by SPI1 and KLF4 were significantly elevated in macrophages from NACT-ESCC patients. These findings provide a basis for further exploring myeloid diversity in SA-ESCC and NACT- ESCC.

Vital roles of myeloid in immunotherapy have been reported [29, 30]. Further studies should investigate distributions of immune checkpoints in monocytes and macrophages. Figure 5F and Supplementary Fig. 6B show that expressions of PDCD1LG2 (PD1-L2), CD274 (PD-L1), CD276 (B7-H3), and LAG3, which are associated with suppressed CD8+ T cell activities, were elevated in macrophages when compared to monocytes (Fig. 5F). Expressions of pro-inflammatory related immune checkpoint genes (IL1A and IL6) were significantly elevated in monocytes, compared to macrophages. These markers are potential immunotherapeutic targets in myeloid cells. Therefore, there is a significant heterogeneity in immunotherapeutic responses in myeloid cells and in potential targets for immunotherapy in myeloid cells.

### Chemotherapy altered the role of plasma B cells in the TME

**Fig. 6:**
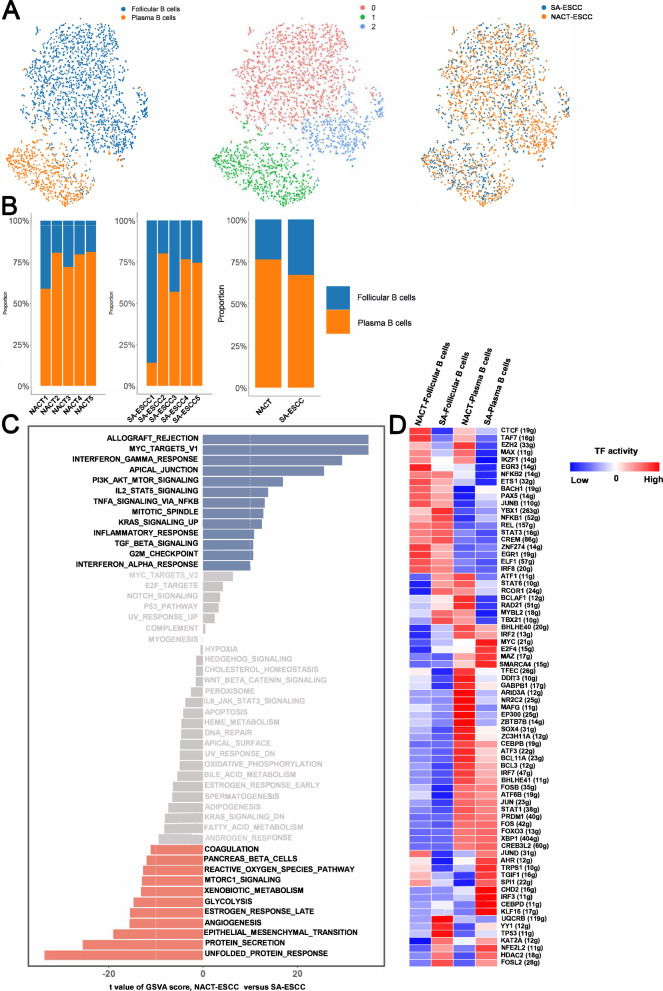
The scRNA Profiles for B cells in SA-ESCC and NACT-ESCC. **A** The TSNE plot of B cells in SA-ESC and NACT-ESCC samples. **B** The proportion of each B cell subtypes in SA-ESCC and NACT-ESCC samples. **C** GSVA analysis in plasma B cells from different conditions. **D** Heatmap showing the activity of TFs in each B cell subtypes in each condition. The TF activity is scored using AUCell.

Based on the expressions of CD79A and IGKC, a total of 2999 B cells were selected for downstream analysis. After re-clustering these cells, a total of three sub-clusters were identified (Fig. 6A). Based on known markers, a total of two sub-cell types were confirmed in B cells (Supplementary Fig. 7A).

Plasma B cells (2198) represented the most common B cell type in SA-ESCC and NACT-ESCC. Notably, relative proportions of plasma B cells were increased in NACT-ESCC compared to SA-ESCC (Fig. 6B). Pathway analysis revealed that interferon response-related pathways, MYC, and allograft rejection pathways were enriched in plasma B cells derived from NACT-ESCC. Besides, unfolded protein responses and angiogenesis pathways were upregulated in plasma B cells from SA-ESCC (Fig. 6C). Potential regulators underlying gene expression differences in plasma B cells or follicular B cells from SA-ESCC and NACT-ESCC patients were determined through SCENIC analysis. Expression levels of genes regulated by STAT3 were elevated in follicular B cells, with higher levels observed in follicular B cells from SA-ESCC samples, whereas high expression levels of ATF3 were observed in plasma B cells, especially in plasma B cells derived from NACT-ESCC patients (Fig. 6D and Supplementary Fig. 7B). Notably, we also found that gene expression levels of HMG20A can be modulated by STAT3. These findings show the complex regulatory network of B cells in NACT-ESCC and SA-ESCC patients.

### Significant differences in CD8+ T cell levels were observed between NACT-ESCC and ESCC patients

**Fig. 7:**
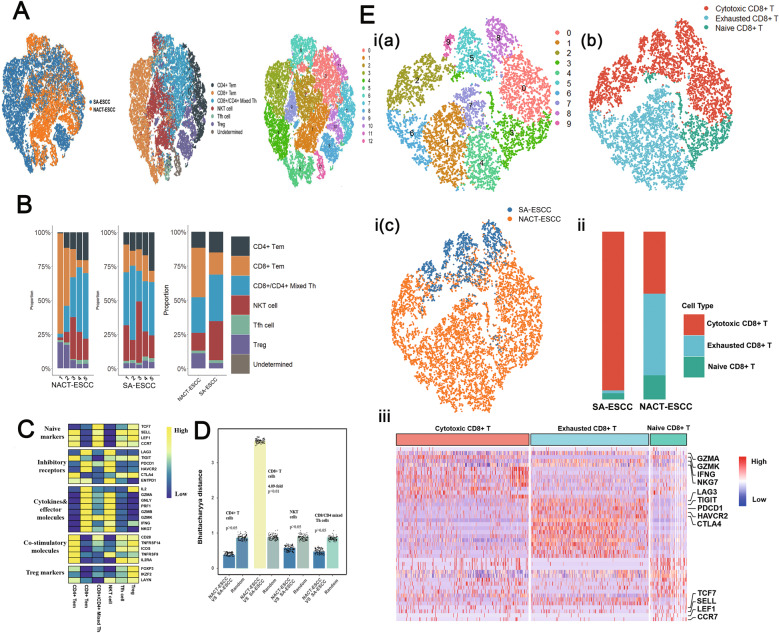
The scRNA Profiles for T cells in SA-ESCC and NACT-ESCC. **A** The TSNE plot of T cells in SA-ESC and NACT-ESCC samples. **B** The proportion of each T cell subtypes in SA-ESCC and NACT-ESCC samples. **C** Average expression of selected T cell function-associated genes of naïve markers, inhibitory receptors, cytokines and effector molecules, co-stimulatory molecules, and Treg markers in each T cell subtype. **D** Quantification of differences between major T cell subtypes in NACT-ESCC and SA-ESCC. Each dot stands for a subsample of 500 cells from PCA space for NACT-ESCC and SA-ESCC or a sample of 500 cells from a random group. The height of the bar is the mean of the subsample. **E** The overview of CD8+ T cells. I, the TSNE plot of CD8+ T cells with each colored by its clusters (a), the associated cell type (b), and sample type of origin (SA-ESCC or NACT-ESCC) (c). II, the proportion of each CD8+ T cell subtypes in SA-ESCC and NACT-ESCC samples. III, heatmap of marker genes of each CD8+ T cell subtypes.

Based on the expressions of CD3D and CD3E, a total of 36,706 T cells were selected. After re-clustering these cells, ten sub-clusters were identified. A total of six sub-cell types were identified based on expressions of known markers (Fig. 7A).

T cells represented the most prevalent immune cell types in SA-ESCC and NACT-ESCC (Fig. 1C). Compared to T cell subtypes in SA-ESCC, CD8+ T cell levels were significantly elevated in NACT-ESCC patients. In addition, suppressed levels of NKT cells, CD8/CD4 mixed Th, and CD4+ T cells were observed in NACT-ESCC (Fig. 7B and Supplementary Fig. 7C). T cell function-associated genes were selected to evaluate functional statuses for each T cell subtype. Cytokines, effector molecules and inhibitory receptors were highly expressed in CD8+ and NK T cells, implying that cytotoxic and inhibitory statuses coexist in CD8+ T and NK T cells. Further, CD4+ T cells exhibited relatively elevated expressions of naïve and co-stimulatory molecule markers (Fig. 7C).

Bhattacharyya distance was used to measure the similarity of two probability distributions and for the estimation of similarities of T cell subtypes between NACT-ESCC and SA-ESCC (“Methods” detailed). Differences between NACT-ESCC and ESCC were found in CD8+ T cells (with 4.11-fold change, *p* < 0.001) (Fig. 7D). Re-clustering CD8+ T cells resulted in 10 clusters and three sub CD8+ T cells, including cytotoxic CD8+ T cells, exhausted CD8+ T cells, and naïve CD8+ T cells (Fig. 7E). Levels of exhausted CD8+ T cells and naïve CD8+ T cells were significantly elevated in NACT-ESCC, implying that chemotherapy alters the TME of ESCC. To validate the activation/exhaustion status of CD8+ T cells between NACT-ESCC and ESCC patients, flow cytometry was performed in our study. Consequently, we found the proportion of CD8+LAG3+PD-1+ T cells in NACT-ESCC is remarkably higher, while the proportion of CD8+CD69+CD25+ T cells were significantly higher in SA-ESCC. (Supplementary Fig. 8A). Overall, our study revealed the different activation/exhaustion status of CD8+ T cells between NACT-ESCC and ESCC patients.

**Fig. 8:**
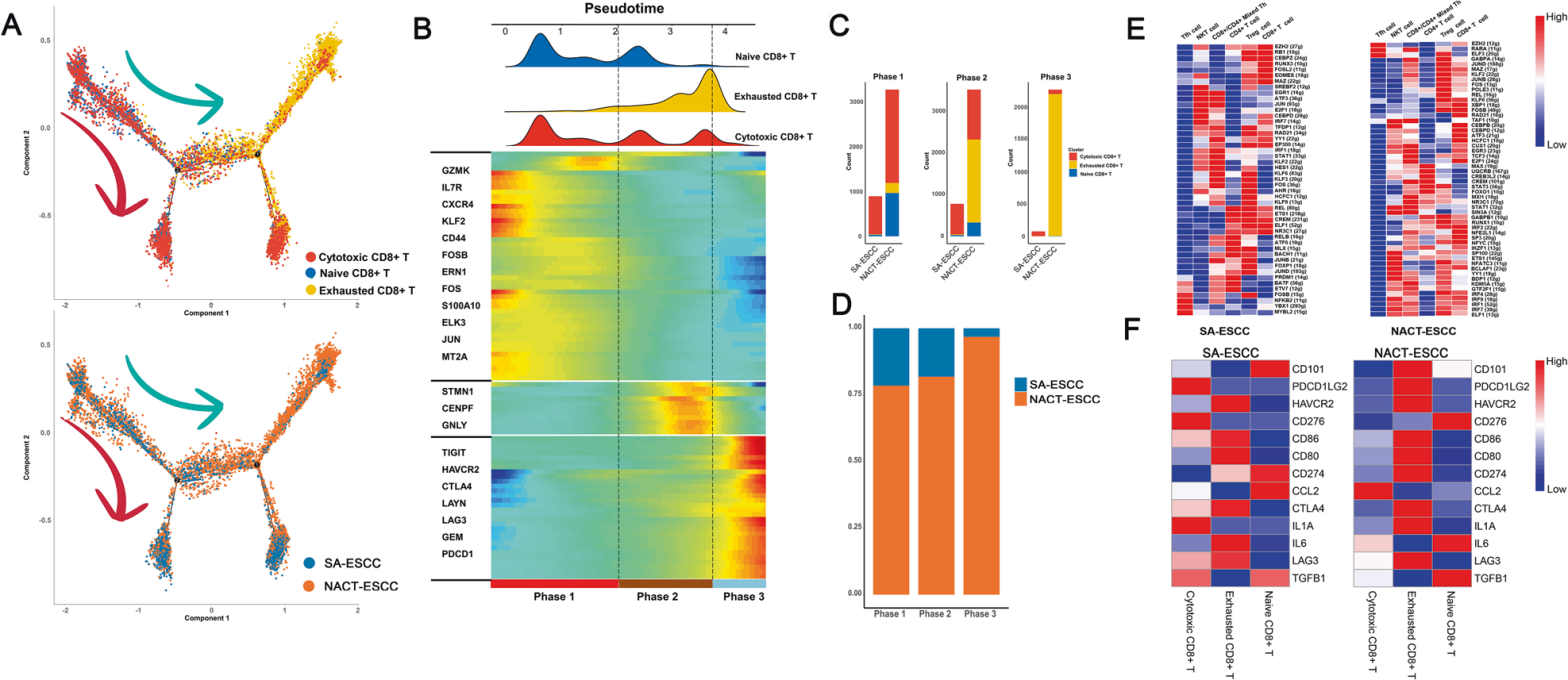
The scRNA Profiles for CD8+ T cells in SA-ESCC and NACT-ESCC. **A** Potential developmental trajectory of CD8+ T cells inferred by analysis with Monocle2. **B** Dynamic changes in gene expression of CD8+ T cells during the transition (divided into 3 phases), subtypes are labeled by colors (upper panel). **C** Histogram showing the cell distribution of CD8+ T cells, in SA-ESCC and NACT-ESCC samples. CD8 subtypes labeled by colors. **D** Histogram showing the cell distribution of SA-ESCC and NACT-ESCC samples. **E** Heatmap showing the activity of TFs in each CD8+ T cell subtypes in each condition. The TF activity is scored using AUCell. Left, SA-ESCC. Right, NACT-ESCC. **F** Heatmap showing the activity of immune checkpoints in each T cell subtypes in each condition. Left, SA-ESCC. Right, NACT-ESCC.

Differentiation trajectory analysis revealed that CD8+ T cells exhibited a branched structure, starting with naïve CD8+ T and cytotoxic CD8+ T cells bifurcating into either cytotoxic CD8+ T cells or exhausted CD8+ T cells (Fig. 7E). Then, to reveal functional alterations of CD8+ T cell populations during pseudotime, transcriptional changes associated with transitional states were investigated, and three phases were identified in CD8+ T cell populations (Fig. 8A, B). Naïve CD8+ and cytotoxic CD8+ T cells were predominantly phase 1 cells, expressing elevated GZMK and IL7R as well as suppressed HAVCR2 and LAG3 levels. Cells in phase 2 exhibited elevated expression levels of STMN1, CENPE, and GNLY (Fig. 8B). Interestingly, phase 2 included cells from exhausted CD8+ and cytotoxic CD8+ T cell populations. Besides, phases 3 (characterized by exhaustion-related genes) were mainly composed of cells from exhausted CD8+ T cells. Additionally, CD8+ T cells derived from SA-ESCC patients mainly belonged to phase 1 and phase 2, with only a few cells assigned to phase 3 (Fig. 8C, D). Conversely, for CD8+ T cells from NACT-ESCC patients, with extended pseudotime, exhausted CD8+ cells gradually increased while cytotoxic CD8+ T and naïve CD8+ T populations gradually decreased (Fig. 8C,D). In summary, although similar transition trajectories were observed in CD8+ T cells from NACT-ESCC and SA-ESCC samples, different immune and transcriptional states were detected, indicating that different immunotherapeutic strategies should be considered for the treatment of SA-ESCC and NACT-ESCC patients.

SCENIC analysis was performed to identify the potential TFs implicated in regulation of different subtypes (Fig. 8E and Supplementary Fig. 8B). E2F1 (E2F transcription factor 1) was found to be upregulated in CD8+ T cells from NACT-ESCC (Fig. 8E). ELF1, JUNB, FOSB were found to be upregulated in CD8+ T cells from NACT-ESCC, whereas in SA-ESCC, these TFs were downregulated (Fig. 8E).

In this study, LAG3 and HAVCR2 were correlated with T cell activity and were highly expressed in exhausted CD8+ T cells in both NACT-ESCC and SA-ESCC (Fig. 8F and Supplementary Fig. 8C). Several differences in expressions of immune checkpoint molecules were observed between NACT-ESCC and SA-ESCC. For example, elevated levels of PDCD1LG2 were observed in exhausted CD8+ T cells in NACT-ESCC, whereas high expresseion levels of PDCD1LG2 were observed in cytotoxic CD8+ T cells in SA-ESCC conditions. These findings imply a significant heterogeneity in immunotherapeutic responses in SA-ESCC and NACT-ESCC.

### Complex communication networks were identified in SA-ESCC and NACT-ESCC conditions

The detailed results can be found in supplementary materials (Supplementary Fig. 9, Supplementary Fig. 10, Supplementary Fig. 11, and Supplementary Fig.12).

## Discussion

In recent years, the scRNA-seq technology has been used to elucidate on the mechanisms underlying ESCC at the single-cell level. Zheng et al. [31] performed scRNA-seq in human ESCC samples and revealed a complex immunosuppressive TME, with CD8 T cells showing a continuous progression from pre-exhausted to exhausted T cells. Yao J et al. [32] used single-cell analysis to investigate cell transition states of ESCC in a mouse model and suggested that suppression of anticancer immune responses promote tumorigenesis and cancer progression. Unlike previous studies, our study performed scRNA analysis for patients with or without preoperative chemotherapy. A total of 113,581 cells were obtained from ESCC patients who had been subjected to surgery alone and neoadjuvant-chemotherapy ESCC samples to represent the cellular landscape of SA-ESCC and NACT-ESCC. Cellular and molecular shifts of malignant cells and related TME for SA-ESCC and NACT-ESCC were characterized, to provide information on the management of ESCC patients.

Changes in glycolytic pathways are closely correlated with tumor regression grade and the prognosis of esophageal cancer after NACT. Low total lesion glycolysis ratio before and after NACT, which reflects suppressed tumor activities of glycolysis, was associated with favorable pathological tumor regression grade and better survival outcomes after NACT and subsequent esophagectomy in ESCC patients [33, 34]. Furthermore, relative metabolic tumor burden is significantly associated with residual lymph node status after neoadjuvant chemoradiotherapy in locally advanced esophageal cancer [35]. In this study, metabolic pathways associated with energy supply (e.g., OXPHOS and Glycolysis) were significantly upregulated in SA-ESCC malignant cells compared to NACT-ESCC malignant cells. OXPHOS, a significant metabolic program in cancer stem cells, is essential for cellular energy and metabolism in tumors [36–38]. Multiple chemotherapy-resistant cancer cells have elevated OXPHOS functions [39–41]. Moreover, we also found the hypoxia-related genes are downregulated in NACT-ESCC, which indicated pathways closely associated with energy supply are promising targets for the treatment of chemotherapy-resistant patients.

It has been revealed that chemotherapeutic agents have the capability to alter the immune composition in the TME [42]. In our study, we observed levels of exhausted CD8+ T cells were significantly elevated in NACT-ESCC, which was concordant with the findings of previous studies [43, 44]. In addition, Christina and colleagues [45] revealed a significantly higher density of CD8+ T cells and a significantly lower density of FOXP3+ T cells in the primary tumors after NACT. In breast cancer, it was reported that the presence of disseminated tumor cells after NACT was significantly associated with higher levels of CD4+ T cells located in the tumor center pre-NACT [46]. NACT in gastric cancer induced CD3+ and CD8+ T lymphocytes as well as CD68+ and CD163+ macrophages in the tumor microenvironment in combination with its direct cytotoxic effects [47]. In conclusion, it was shown that NACT could have a significant impact on the tumor microenvironment, mostly with changed lymphocytic infiltration and PD-L1 expression in certain patients, which helped to predict the patient’s reaction to NACT as well as their prognosis. However, most studies focus on the results of immune changes caused by NACT, and whether this is attributable to cytotoxicity-induced antigen release in these typically chemo-sensitive tumors is unknown, which means the mechanism remains to be further explored.

On the other hand, in our study, we also revealed that the NACT-ESCC group exhibited increased plasma B and monocyte levels when compared to the SA-ESCC group.

Previous studies had reported that plasma B cells are found scattered at tumor margins, close to cancer-associated fibroblasts (CAFs), in tumor-associated aggregates, and in unorganized clusters or organized tertiary lymphoid structures (TLS), playing a key role in responses to checkpoint blockade immunotherapies [48, 49]. For example, plasma B cells high group was found to respond better to PD1 blockade therapy, mainly because immune checkpoint blockade might target plasma B cells, since PD-1, PD-L1, CTLA-4, and the B7 molecules are expressed on plasma B cells [50]. Given these findings, recent reviews have identified a key role for plasma B cells in oncology and patient outcomes [51, 52]. Additionally, a central role in cancer-related inflammation is played by cells of the monocyte-macrophage lineage [53]. Furthermore, Macrophages express the ligands for checkpoint molecules, including PD-L1, PD-L2, B7H4 and the CTLA4 ligands B7-1 and B7-2 [54]. As a result, monocytes are known to be a strong predictor of progression-free survival in response to anti-PD-1 immunotherapy [55].

Accumulating evidence has shown that the interaction between cancer cells and the TME influences the sensitivity of cancer cells to chemotherapy. This study revealed that stromal cells were greatly reduced whereas immune cells were markedly elevated after receiving preoperative chemotherapy. Features of the tumor-associated stroma, such as the tumor-stroma ratio, were found to affect patients’ prognosis and clinical outcomes [56]. Besides, the differential chemo-sensitivity caused by stroma organization may be modulated by the qualitative aspects of cells in the stromal tumor microenvironment [56, 57]. Overall, these results reveal new therapeutic targets for ESCC treatment.

This study had some limitations. First, only patients who had undergone preoperative chemotherapy and not preoperative chemoradiotherapy were included in the study. Second, since tumor samples are very heterogeneous, the ten patients included in this study might not be representative of all patients with ESCC. Third, most patients were diagnosed by endoscopy before neoadjuvant chemotherapy, hence there was no enough specimens for scRNA sequencing. However, this study provides a comprehensive analysis of the cellular and molecular landscape of SA-ESCC and NACT-ESCC. In addition, these findings provide a basis for further studies on molecular and cellular therapeutic targets for ESCC treatment.

## Materials and methods

The detailed materials and methods can be found in supplementary materials.

## Supplementary information

Supplementary Materials

Supplementary legends

Supplementary Table 1

Supplementary Figure 1

Supplementary Figure 2

Supplementary Figure 3

Supplementary Figure 4

Supplementary Figure 5

Supplementary Figure 6

Supplementary Figure 7

Supplementary Figure 8

Supplementary Figure 9

Supplementary Figure 10

Supplementary Figure 11

Supplementary Figure 12

## Data Availability

The data used in this study can be obtained by request to C.Z. (czhan10@fudan.edu.cn).

## Code availability

The code used in this study can be obtained by request to C.Z. (czhan10@fudan.edu.cn).
