# Supplementary material for: Dissecting the single-cell transcriptome network in patients with esophageal squamous cell carcinoma receiving operative paclitaxel plus platinum chemotherapy
Source: Oncogenesis. 2021 Oct 26;10(10):71. doi: 10.1038/s41389-021-00359-2 (PMC8546051; doi:10.1038/s41389-021-00359-2)
Supplement: Supplementary file 1 — Supplementary Materials [file 41389_2021_359_MOESM1_ESM.docx]

**Dissecting the Single-Cell Transcriptome Network In Patients with Esophageal Squamous Cell Carcinoma Receiving Operative Paclitaxel Plus Platinum Chemotherapy**

Zhencong Chen^1#^, YiWei Huang^1#^, Zhengyang Hu^1#^, Mengnan Zhao ^1#^, Yunyi Bian^1^, Zongwei Chen^1^, Yuansheng Zheng^1^, Guoshu Bi^1^, Yanrui Pang^2^, Cheng Zhan^1*^, Zongwu Lin^1*^, Weigang Guo^1*^, Qun Wang^1^, Lijie Tan^1^

^1^ Department of Thoracic Surgery, Zhongshan Hospital, Fudan University, No. 180, Fenglin Road, Shanghai, 200032, China

^2^ Department of Pathology of Zhongshan Hospital, Fudan University, No. 180, Fenglin Road, Shanghai, 200032, China

^#^These authors contributed equally: Zhencong Chen, YiWei Huang, Zhengyang Hu, and Mengnan Zhao.

*The authors are all corresponding authors of this paper.

**Corresponding authors’ information**

Cheng Zhan & Zongwu Lin & Weigang Guo, Department of Thoracic Surgery, Zhongshan Hospital, Fudan University, No. 180, Fenglin Road, Shanghai, 200032, China;

**Phone:** 86-21-64041990; Fax: 86-21-64041990;

**Email:** czhan10@fudan.edu.cn;

lin.zongwu@zs-hospital.sh.cn;

guo.weigang@zs-hospital.sh.cn

**Materials and Methods**

**Patient and tissue sample collection**

Ten patients (five undergoing neoadjuvant-chemotherapy ans five undergoing surgery) who were diagnosed with esophageal squamous cell carcinoma (ESCC) at the Department of Thoracic Surgery, Zhongshan Hospital, Fudan University (FDZSH) were included in this study. Additional five SA-ESCC samples, corresponding five non-malignant samples, and five NACT-ESCC samples were obtained to perform flow cytometry and qRT-PCR analysis.

Patients in the neoadjuvant-chemotherapy ESCC group were treated as follows: (1) preoperative chemotherapy consisting of paclitaxel and cisplatin: 135 mg/m2 paclitaxel on day 1 and 75 mg/m2 cisplatin on day 1 through intravenous drip infusion, a second cycle was given after 3 weeks; (2) after receiving two courses of chemotherapy, patients underwent minimally invasive esophagectomy; (3) the interval between preoperative chemoradiotherapy and surgery was 4-8 weeks.

**Sample preparation**

Fresh ESCC samples were mechanically dissociated and enzymatic digested. Tissues were minced into small pieces and mixed with enzyme H, enzyme A, enzyme R, and Dulbecco's Modified Essential Medium for 30min at 37℃ in a gentle MACS C Tube. Subsequently, a 40-um nylon mesh, Red Blood Cell Lysis Solution (10×) (Sigma-Aldrich, St. Louis, MO, USA), and Dead Cell Removal Kit (Miltenyi Biotec) were used to filter the digested cells.

**Single-cell RNA sequencing and read processing**

Single Cell A Chip Kit, Single Cell3′ Library and Gel Bead Kit V2, and i7 Multiplex Kit (10x Genomics, Pleasanton, CA, USA) were used for single-cell sequencing according to the manufacturer's instructions.

A library was prepared with the 10X Chromium single-cell kit. The libraries were sequenced using Illumina sequencing platform (HiSeq X Ten; Illumina, San Diego, CA, USA) and mapped onto the GRCh38 human reference genome using Cell Ranger toolkit (version 2.1.0).

**The 10x scRNA-seq data analysis**

To ensure high-quality cells, raw count data was subjected to quality control to filter cells before data analysis. The quality quality control measures included: 1) number of expressed genes lower than 200 or larger than 5000; 2) >10% of UMIs were mapped onto mitochondrial or ribosomal genes; 3) genes with low expression levels (with min.cells < 0.1% cells).

Next, the Seurat R package ^1^ was used to analyze scRNA-seq data. The functions, "CreateSeuratObject" and "FindVariableFeatures" were utilized to create a Seurat object and identify the top 2,000 highly variable genes, respectively. Unsupervised dimensional reduction and clustering analysis were performed using "RunPCA," "FindClusters," and RunTSNE functions based on highly variable genes. In addition, the gene expression markers of all identity classes were determined using the "FindAllMarkers" function. The identity classes were annotated using the SingleR package^2^, CellMarker dataset^3^, and previous studies^4,5^. The "SubsetData" function was utilized in subclusters analysis to select cells, and sub-clusters were annotated based on dominant expression cell markers.

**Prediction of cell types as a tumor or normal epithelium**

The R package scCancer^6^, which is based on “infercnv”^7^, was used to distinguish between normal from tumor epithelium cells. To estimate copy number variations (CNVs), Seurat object of epithelium cells was used as the input file, while cells except epithelium cells were used as reference data.

The R package scPred was used to validate the reliability of cell-type classification as described in previous studies ^8,9^. A training dataset was built using half of the epithelial cells with a seed of 0.51 using the "createDataPartition" function. Using the "EigenDecompose," "getFeatureSpace," and "trainModel" functions, PCA analysis conducted to obtain the most cell type-informative principal components, for the construction of the training model, respectively. Cell types of remaining cells were determined using the "scPredict" (threshold was 0.6) and "getPredictions" functions.

**Quantification of differences between cells in neoadjuvant-chemotherapy ESCC and ESCC samples**

Based on Bhattacharyya distance ^10,11^, differences between cells in NACT-ESCC and SA-ESCC samples were explored. Cell subtypes having more than 500 cells under SA-ESCC and NACT-ESCC conditions were used for distance measurement. In addition, the top 2000 highly variable genes were selected for downstream analysis using "FindVariableFeatures" function in Seurat. Thereafter, PCA analysis was performed for the cells that met the filtering criteria and the top 50 PCs were selected for subsequent calculation. A total of 500 cells were randomly selected from SA-ESCC or NACT-ESCC conditions for 100 times. Finally, the Bhattacharyya distance was estimated using the following formulae:

$$D_{\mathrm{Bhatta}}=\frac{1}{8}\left( \vec{u_{1}}-\vec{u_{2}} \right)^{T}\Sigma^{-1}\left( \vec{u_{1}}-\vec{u_{2}} \right)+\frac{1}{2}\log_{e} (\frac{|\Sigma|}{\sqrt{|\Sigma_{1}||\Sigma_{2}|}})$$

Where $\vec{u_{1}}$ and$\vec{u_{2}}$ are the mean vectors of each distribution, and $\Sigma=(\Sigma_{1}+\Sigma_{2})/2$. Moreover, Bhattacharyya distance between cells randomly selected from the same conditions (NACT-ESCC or SA-ESCC) was estimated to serve as background distribution. Statistical significance between cells from the two groups was determined using Wilcoxon rank-sum test.

**Gene Set Variation Analysis and Functional enrichment analysis**

The GSVA package was used for Gene Set Variation Analysis (GSVA) ^12^. Gene sets of pathways were retrieved from Molecular Signatures Database (MSigDB) database (<http://software.broadinstitute.org/gsea/msigdb/index.jsp>) ^13^. The Metascape (<http://metascape.Org>) database ^14^ was used for Gene Ontology (GO) and Kyoto Encyclopedia of Genes and Genomes (KEGG) pathway functional enrichment analysis. P < 0.01 was considered statistically significant and the threshold for number of enriched genes was set at > 3.

**SCENIC analysis**

SCENIC analysis was performed using the "SCENIC" R package ^15^. Motif databases (hg19-500bp-upstream-7species.mc9nr.feather and hg19-tss-centered-10kb-7species.mc9nr.feather) were retrieved from cisTarget databases (https://resources.aertslab.org/cistarget/databases) for RcisTarget and GRNboost. "AUCell," "RcisTarget," and "GENIE3" R packages were used for SCENIC analysis^16^. The threshold of target gene was: ‘HighConfAnnot’= ‘True’ and ‘Genie Weight’= top 10%.

**Trajectory analysis**

Trajectory analysis was performed using the R package monocle2 to explore tumor‐reprogramming processes in single cells as described previously^17^. A monocle subject was built using "newCellDataSet" function based on differentially expressed genes. The "ReduceDimension" and "orderCells" functions were then used for reduction of dimensions and to place cells onto a pseudotime trajectory.

**Analyses of metabolic pathways**

The metabolic activities of cell types were estimated as described byprevious study. ^18^. Analysis of the metabolic program was based on mean expression level of a metabolic gene across cell types.

**Cell-cell communications**

The CellPhoneDB Python package (1.1.0)^19^ was used to explore potential communications among different cell types in single-cell data. Data was normalized using Seurat package and used as the input file. Receptors and ligands expressed in more than 10% of the cells were included in the analysis. Ligands and receptor pairs with P-value >0.05 were discarded.

The signaling communications among different cell types were analyzed using the CellChat^20^ R package. A CellChat object was constructed using the "CreateCellChat" function, after which the "computeCommunProb" and "computeCommunProbPathway" functions were utilized for calculation of communication probability. The number of patterns was set as five.

**Flow Cytometry and qRT-PCR**

Cell samples were lysed and non-specific antibodies were blocked with a phosphate buffer containing 20 μg/mL human IgG and 3% fetal bovine serum for 15 minutes. Next, cells and BV510-conjugated anti-human CD45 (5 μL/106 cells; cat. no.: 563204, BD Biosciences), PerCP-Cy5.5-conjugated anti-human CD8 (5 μL/106 cells; cat. no.: 560662, BD Biosciences), PE-Cyanine7-conjugated anti-human CD223(LAG-3) (5 μL/ 106 cells; cat. no.: 2061237, eBioscience), FITC-conjugated anti-human CD279 (5 μL/ 106 cells; cat. no.: 367433, Biolegend), PE-conjugated anti-human CD25 (5 μL/ 106 cells; cat. no.: 302605, Biolegend), PE/Dazzle 594-conjugated anti-human 279 (5 μL/ 106 cells; cat. no.: 367433, Biolegend), or BV421-conjugated anti-human CD69 (5 μL/106 cells; cat. no.: 310929, BD Biosciences) were incubated on ice for 30 minutes.

The required cells were quantified and isolated using FACSAria III (BD Biosciences) and FlowJo software (TreeStar, Woodburn, OR, USA) was employed to analyze results. Once isolated, the non-malignant cells expressing KRT5 and tumor cells expressing EPCAM, RNA was extracted and then reverse-trascribed into cDNA using the kit (Illumina, San Diego, USA). Finally, the QuantStudio 6 Flex (Thermo Fisher Scientific) was employed for sequencing. Comparison was performed by Wilcoxon test in flow cytometry and qRT-PCR.

**Immunofluorescence**

Immunofluorescence (IF) specific for APOE (Rabbit, AF5178, Affinity Biosciences, RRID: AB_2837664) and SPP1 (Rabbit, AF0227, Affinity Biosciences, RRID: AB_2833402) was performed as previously described. Briefly, the paraffin-embedded slides were dewaxed and rehydrated. After antigen retrieval, block of endogenous peroxidase activity and non-specific antigens, and incubation with primary antibodies and horseradish peroxidase-conjugated secondary antibody, the slides were incubated with Opal tyramide signal amplification (TSA) Fuorochromes (Opal 2-Color Manual IHC Kit, G1236, Servicebio Co., Ltd) for 10 min at room temperature. After the second run, the slides were stained with DAPI. Immunofluorescence was quantified using ImageJ (ver. 1.32j, NIH). Comparison was performed by one-way ANOVA in immunofluorescence.

**Statistical analysis**

The statistical tools, methods, and thresholds used for data analysis are explicitly described and the results or other details are given in the figure legends or Materials and Methods. The statistical significances of groups are represented as *p < 0.05, ** p < 0.01, *** p < 0.001, and **** p < 0.0001.

**Results**

**Complex communication networks were identified in SA-ESCC and NACT-ESCC conditions**

The 'CellChat' ^20^ function was used to identify ligand-receptor pairs and molecular interactions among major cell types. More ligand-receptor pairs were identified in NACT-ESCC compared to SA-ESCC, implying that complex interactions occur in NACT-ESCC TME. Some ligand-receptor pairs (e.g., MDK and its receptors or SPP1 and its receptors) were highly expressed in both SA-ESCC and NACT-ESCC (Supplementary Fig. 9A and Supplementary Fig. 10). A pattern recognition method was used to identify global communication patterns and critical signals among different cell types to further investigate the communications for individual pathways in each cell group (Methods detailed). Five outgoing and incoming patterns were observed in SA-ESCC and NACT-ESCC, respectively.

The 'CellChat' pattern recognition module was used to detect actual signaling events in SA-ESCC. At the incoming end of signaling, the WNT and ncWNT signals were first secreted by endothelial and malignant cells, and immune cells were mainly enriched with CXCL and CX3C pro-inflammatory signals (Supplementary Fig. 9B). At the outcoming end of signaling, immune cells were driven by 2, 3, and 5 patterns, including IL1 and IL10, while malignant cells were dominated by SEMA3, BMP, and FGF in SA-ESCC (Supplementary Fig. 9B).

Outgoing and incoming T cells in NACT-ESCC were enriched in pattern 5, representing multiple pathways, including CCL, IL2, and OX40 (Supplementary Fig. 9B). In addition, outgoing and incoming malignant and non-malignant cells that were characterized by pattern 1 were identified, representing pathways such as WNT, BMP, NT, and IGF. Most myeloid cells were found to be dominated by pattern 2 in outgoing and incoming patterns driven by IFN-II, MIF, CSF, BAFF, and SPP1. We further investigated the role of each cell type in IL2, WNT, and SPP1 (Supplementary Fig. 11). Immune cells, especially T cells, were the most prominent sources of the IL2 signaling network in both autocrine and paracrine signaling. Immune cells were the main sources of paracrine signaling in the Wnt signaling network compared to the IL2 signaling network. Moreover, malignant and myofibroblast cells significantly contributed to Wnt signaling. Macrophages were dominant sources of SPP1 in SPP1 signaling pathway.

To investigate the functional status of these pathways in NACT-ESCC and SA-ESCC patients, we clustered and mapped them onto a shared two dimensional manifold. Supplementary Fig. 12A shows that a total of four pathway groups were identified. Clusters 2 and 3 mainly included growth factor pathways such as PDGF, NGF, and FGF. In contrast, inflammation-related pathways, including CXCL, IL10, and IFN-II pathways were detected in clusters 1 and 4. Some signaling pathways (e.g. ncWNT, OX40 and SPP1) were identified in the same clusters, implying that these pathways play important roles in the tumorigenesis of ESCC. Moreover, some pathways (e.g. IL-2, CXCL and WNT) were assigned to different clusters, suggesting that these pathways play different roles in NACT-ESCC and SA-ESCC. Evaluation of euclidean distance between signaling pathways in NACT-ESCC and SA-ESCC revealed that IL-2, CXCL and WNT had a greater distance while ncWNT, OX40 and SPP1 had a lesser distance (Supplementary Fig. 12B).

**Reference：**

1. Macosko, E.Z., Basu, A., Satija, R., Nemesh, J., Shekhar, K., Goldman, M., Tirosh, I., Bialas, A.R., Kamitaki, N., Martersteck, E.M.*, et al.* Highly Parallel Genome-wide Expression Profiling of Individual Cells Using Nanoliter Droplets. *Cell* **161**, 1202-1214 (2015).

2. Aran, D., Looney, A.P., Liu, L., Wu, E., Fong, V., Hsu, A., Chak, S., Naikawadi, R.P., Wolters, P.J., Abate, A.R.*, et al.* Reference-based analysis of lung single-cell sequencing reveals a transitional profibrotic macrophage. *Nature Immunology* **20**, 163-172 (2019).

3. Zhang, X., Lan, Y., Xu, J., Quan, F., Zhao, E., Deng, C., Luo, T., Xu, L., Liao, G., Yan, M.*, et al.* CellMarker: a manually curated resource of cell markers in human and mouse. *Nucleic Acids Research* **47**, D721-D728 (2018).

4. Madissoon, E., Wilbrey-Clark, A., Miragaia, R.J., Saeb-Parsy, K., Mahbubani, K.T., Georgakopoulos, N., Harding, P., Polanski, K., Huang, N., Nowicki-Osuch, K.*, et al.* scRNA-seq assessment of the human lung, spleen, and esophagus tissue stability after cold preservation. *Genome biology* **21**, 1 (2019).

5. Lambrechts, D., Wauters, E., Boeckx, B., Aibar, S., Nittner, D., Burton, O., Bassez, A., Decaluwe, H., Pircher, A., Van den Eynde, K.*, et al.* Phenotype molding of stromal cells in the lung tumor microenvironment. *Nature medicine* **24**, 1277-1289 (2018).

6. Guo, W., Wang, D., Wang, S., Shan, Y. & Gu, J. *scCancer: a package for automated processing of single cell RNA-seq data in cancer*, (2019).

7. Puram, S.V., Tirosh, I., Parikh, A.S., Patel, A.P., Yizhak, K., Gillespie, S., Rodman, C., Luo, C.L., Mroz, E.A., Emerick, K.S.*, et al.* Single-Cell Transcriptomic Analysis of Primary and Metastatic Tumor Ecosystems in Head and Neck Cancer. *Cell* **171**, 1611-1624.e1624 (2017).

8. Sathe, A., Grimes, S.M., Lau, B.T., Chen, J., Suarez, C., Huang, R.J., Poultsides, G. & Ji, H.P. Single-Cell Genomic Characterization Reveals the Cellular Reprogramming of the Gastric Tumor Microenvironment. *Clinical cancer research : an official journal of the American Association for Cancer Research* (2020).

9. Alquicira-Hernandez, J., Sathe, A., Ji, H.P., Nguyen, Q. & Powell, J.E. scPred: accurate supervised method for cell-type classification from single-cell RNA-seq data. *Genome biology* **20**, 264 (2019).

10. Cillo, A.R., Kurten, C.H.L., Tabib, T., Qi, Z., Onkar, S., Wang, T., Liu, A., Duvvuri, U., Kim, S., Soose, R.J.*, et al.* Immune Landscape of Viral- and Carcinogen-Driven Head and Neck Cancer. *Immunity* **52**, 183-199.e189 (2020).

11. Mohammadi, A. & Plataniotis, K.N. Improper Complex-Valued Bhattacharyya Distance. *IEEE transactions on neural networks and learning systems* **27**, 1049-1064 (2016).

12. Hänzelmann, S., Castelo, R. & Guinney, J. GSVA: gene set variation analysis for microarray and RNA-seq data. *BMC bioinformatics* **14**, 7 (2013).

13. Hanzelmann, S., Castelo, R. & Guinney, J. GSVA: gene set variation analysis for microarray and RNA-seq data. *BMC bioinformatics* **14**, 7 (2013).

14. Zhou, Y., Zhou, B., Pache, L., Chang, M., Khodabakhshi, A.H., Tanaseichuk, O., Benner, C. & Chanda, S.K. Metascape provides a biologist-oriented resource for the analysis of systems-level datasets. *Nature communications* **10**, 1523 (2019).

15. Aibar, S., Gonzalez-Blas, C.B., Moerman, T., Huynh-Thu, V.A., Imrichova, H., Hulselmans, G., Rambow, F., Marine, J.C., Geurts, P., Aerts, J.*, et al.* SCENIC: single-cell regulatory network inference and clustering. *Nature methods* **14**, 1083-1086 (2017).

16. Luo, T., Zheng, F., Wang, K., Xu, Y., Xu, H., Shen, W., Zhu, C., Zhang, X., Sui, W., Tang, D.*, et al.* A single-cell map for the transcriptomic signatures of peripheral blood mononuclear cells in end-stage renal disease. *Nephrology, dialysis, transplantation : official publication of the European Dialysis and Transplant Association - European Renal Association* (2019).

17. Qiu, X., Mao, Q., Tang, Y., Wang, L., Chawla, R., Pliner, H.A. & Trapnell, C. Reversed graph embedding resolves complex single-cell trajectories. *Nature methods* **14**, 979-982 (2017).

18. Xiao, Z., Dai, Z. & Locasale, J.W. Metabolic landscape of the tumor microenvironment at single cell resolution. *Nature communications* **10**, 3763 (2019).

19. Vento-Tormo, R., Efremova, M., Botting, R.A., Turco, M.Y., Vento-Tormo, M., Meyer, K.B., Park, J.E., Stephenson, E., Polański, K., Goncalves, A.*, et al.* Single-cell reconstruction of the early maternal-fetal interface in humans. *Nature* **563**, 347-353 (2018).

20. Jin, S., Guerrero-Juarez, C.F., Zhang, L., Chang, I., Myung, P., Plikus, M.V. & Nie, Q. Inference and analysis of cell-cell communication using CellChat. 2020.2007.2021.214387 (2020).
