# Supplementary material for: Dissecting the single-cell transcriptome network in patients with esophageal squamous cell carcinoma receiving operative paclitaxel plus platinum chemotherapy
Source: Oncogenesis. 2021 Oct 26;10(10):71. doi: 10.1038/s41389-021-00359-2 (PMC8546051; doi:10.1038/s41389-021-00359-2)
Supplement: Supplementary file 2 — Supplementary legends [file 41389_2021_359_MOESM2_ESM.docx]

**Supplementary Figure 1. ScRNA-seq profiling of the SA-ESCC and NACT-ESCC**

(A) The number of each cell type in SA-ESC and NACT-ESCC samples.

(B) Identified stromal cells and immune cells from SA-ESCC and NACT-ESCC patients by flow cytometry. Comparison was performed by Wilcoxon test.

(C) scPred Analysis to Validate Cell Annotations in Seurat Object. Probabilities for malignant versus non-malignant in the train model. The panel represents a prediction model. Malignant cells are cells except the positive class.

**Supplementary Figure 2. Flow cytometry and qRT-PCR for malignant cells and Non-malignant epithelial cells.**

(A) Identified and sorted the non-malignant epithelial cells, SA-ESCC-malignant epithelial cells, and NACT-ESCC epithelial cells from SA-ESCC, NACT-ESCC, and non-malignant patients by flow cytometry.

(B) The gene expression levels of SOX4 (P < 0.01) and MDK (P <0.01) were significantly higher in SA-ESCC-malignant epithelial cells, KRT14 (P < 0.01) and S100A2 (P <0.01) were significantly higher in non-malignant epithelial cells, MUC4 and WFDC2were significantly in NACT-ESCC epithelial cells. Comparison was performed by Wilcoxon test

**Supplementary Figure 3.** **Functional enrichment analysis for the for stromal cells in SA-ESCC patients (A) and immune cells in NACT-ESCC conditions ( B). Upper: colored by cluster ID, where nodes that share the same cluster ID are typically close to each other. Bottom: colored by p-value, where terms containing more genes tend to have a more significant P-value.**

**Supplementary Figure 4. Expression of marker genes for stromal cells and functional enrichment analysis for immune endothelial cells (immune EDCs)**

(A) Expression of marker genes for endothelial cells.

(B) Expression of marker genes for fibroblasts.

(C) Functional enrichment analysis for immune EDCs in different conditions. I, SA-ESCC. II, NACT-ESCC.

**Supplementary Figure 5. Feature plot of marker genes for myeloid cells and dot plot of marker genes for myeloid cells.**

(A) Feature plot of marker genes for myeloid cells.

(B) Dot plot of marker genes for myeloid cells.

(C) TFs with downstream genes of through network analyses in myeloid cells form SA-ESCC (Left) and NACT-ESCC patients (Right). The thickness of edges in the network denotes the correlation coefficient between TFs and downstream genes. TFs were marked in red circle.

**Supplementary Figure 6. Immunofluorescence of SPP1 and Violin plots of immune checkpoints in myeloid cells**

(A) Immunofluorescence staining of SPP1 in NACT-ESCC (n=5) / SA-ESCC (n=5) tissues. Comparison was performed by one-way ANOVA in immunofluorescence.

(B) Violin plots of immune checkpoints upregulated or downregulated between monocytes and macrophages cells.

**Supplementary Figure 7. The subtypes of B/T cells in ESCC**

(A) Feature plot of marker genes for B cells.

(B) TFs with downstream genes of through network analyses in B cells form SA-ESCC and NACT-ESCC patients. The thickness of edges in the network denotes the correlation coefficient between TFs and downstream genes. TFs were marked in red circle.

(C) Expression of marker genes for T cells. I, Feature plot of CD3D and CD3E. II, Heatmap showing the marker genes for each T cell subtypes.

**Supplementary Figure 8. Flow cytometry and qRT-PCR for T cells.**

(A) Identified and sorted the activated/exhausted CD8+ T cells in SA-ESCC and NACT-ESCC by flow cytometry. Comparison was performed by Wilcoxon test.

(B) TFs with downstream genes of through network analyses in T cells form SA-ESCC and NACT-ESCC patients. The thickness of edges in the network denotes the correlation coefficient between TFs and downstream genes. TFs were marked in red circle.

(C)The gene expression levels of immune checkpoints in CD8+ T cells in in SA-ESCC and NACT-ESCC respectively.

**Supplementary Figure 9. Crosstalk Between Cancer and Immune Cells.**

(A). Overview of selected ligand–receptor interactions of tumor cells and immune cells. P values are presented as circles, the scale to the right explains P-values (permutation test). Assays were carried out at the mRNA level but were extrapolated to infer protein interactions.

Left, SA-ESCC. A, CD4+ T cells; B, CD8+/CD4+ Mixed T helper; C, Cytotoxic CD8+ T; D, NKT cell; E, Regulated T cells; F, DCs; G, Follicular B cells; H, Macrophages; I, Monocytes; J, Mixed−Monocytes.

Right, NACT-ESCC. A, CD4+ T cells; B, CD8+/CD4+ Mixed T helper; C, Cytotoxic CD8+ T; D, naïve CD8+ T cell; E, NKT cell; F, Exhausted CD8+ T cell; G, Regulated T cells; H, Tfh cell; I, DCs; J, Follicular B cells; K, Plasma B Cell; L, Macrophages; M, Monocytes; N, Mixed−Monocytes.

(B). The plot showing outgoing and incoming communication patterns of major cell lineages. I, SA-ESCC. II, NACT-ESCC.

**Supplementary Figure 10. Crosstalk Between Cancer and Stromal Cells.**

Overview of selected ligand–receptor interactions of tumor cells and stromal cells. P values are presented as circles, the scale to the right explains P values (permutation test). Assays were carried out at the mRNA level but were extrapolated to infer protein interactions. SA-ESCC(A), NACT-ESCC(B). A, Myofibroblasts; B, Smooth muscle cells; C, COL14A1 matrix fibroblasts; D, Vascular EDCs; E, Tumor EDCs.

**Supplementary Figure 11. Inferred intercellular communication network and heatmap shows relative importance of each cell group based on computed four network centrality measures of IL2 (A), SPP1 (B), and WNT (C) signaling.**

**Supplementary Figure 12.** **Functional status of pathways in NACT-ESCC and SA-ESCC patients.**

(A) Magnified view of each pathway group.

(B) The overlapping signaling pathways between SA-ESCC and NACT-ESCC were ranked based on their pairwise Euclidean distance in the shared two-dimensions manifold. Larger distance implies larger difference of the communication network between SA-ESCC and NACT-ESCC.

**Supplementary Table 1.** Characteristics of the five SA-ESCC and NACT-ESCC patients.
