# Supplementary material for: Dissecting the single-cell transcriptome network in patients with esophageal squamous cell carcinoma receiving operative paclitaxel plus platinum chemotherapy
Source: Oncogenesis. 2021 Oct 26;10(10):71. doi: 10.1038/s41389-021-00359-2 (PMC8546051; doi:10.1038/s41389-021-00359-2)
Supplement: Supplementary file 3 — Supplementary Table 1 [file 41389_2021_359_MOESM3_ESM.docx]

**Table I Characteristics of the 10 patients included in this study for scRNA-seq analysis**

|  | **Age** | **Sex** | **TNM** | **Smoking status** | **Alcohol units** | **Outcomes** |
| --- | --- | --- | --- | --- | --- | --- |
| SA-1 | 65-70 | Male | T1N1M0G2 | Former | No | Alive |
| SA-2 | 65-70 | Male | T3N0M0G2 | No | Yes | Alive |
| SA-3 | 60-65 | Male | T3N0M0G2 | Former | No | Alive |
| SA-4 | 65-70 | Male | T3N1M0 | Former | No | Alive |
| SA-5 | 50-55 | Male | T2N0M0G2 | Former | Yes | Alive |
| NACT-1 | 55-60 | Male | ypT3N0M0 | Former | No | Alive |
| NACT-2 | 55-60 | Male | ypT3N1M0 | Former | No | Alive |
| NACT-3 | 60-65 | Female | ypT3N2M0 | No | No | Alive |
| NACT-4 | 50-55 | Male | ypT3N0M0 | Former | Yes | Alive |
| NACT-5 | 50-55 | Male | ypT2N0M0 | Former | No | Alive |

**SA：Surgery Alone**

**NACT:** **Neoadjuvant Chemotherapy**
