# Supplementary figures and images for: Dissecting the single-cell transcriptome network in patients with esophageal squamous cell carcinoma receiving operative paclitaxel plus platinum chemotherapy
Source: Oncogenesis. 2021 Oct 26;10(10):71. doi: 10.1038/s41389-021-00359-2 (PMC8546051; doi:10.1038/s41389-021-00359-2)

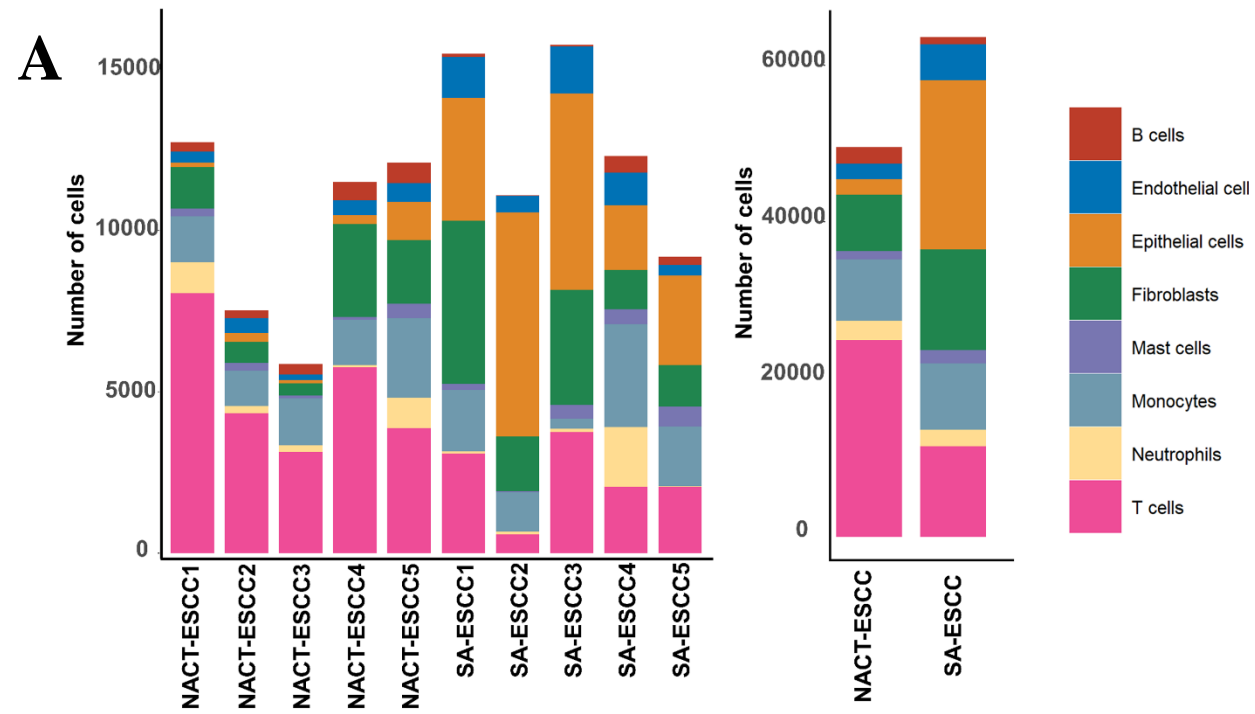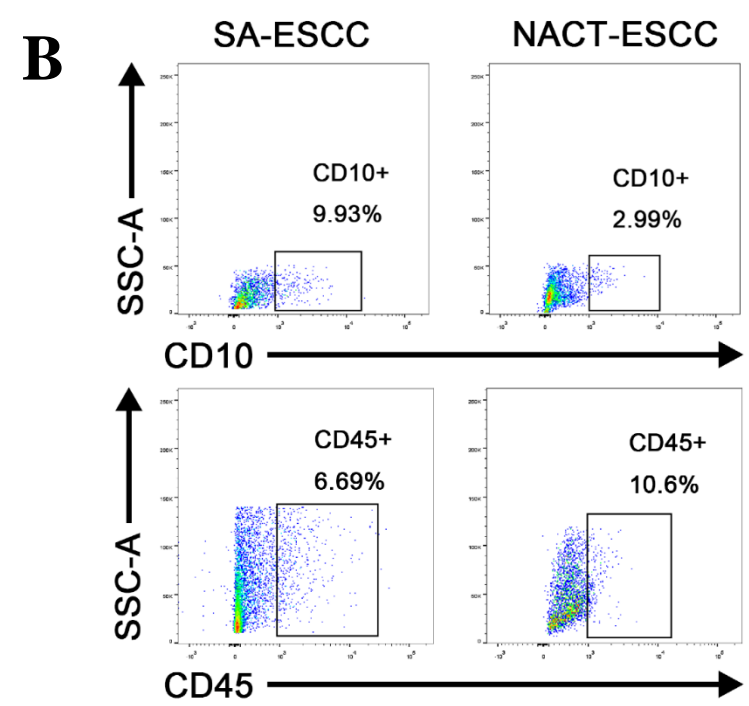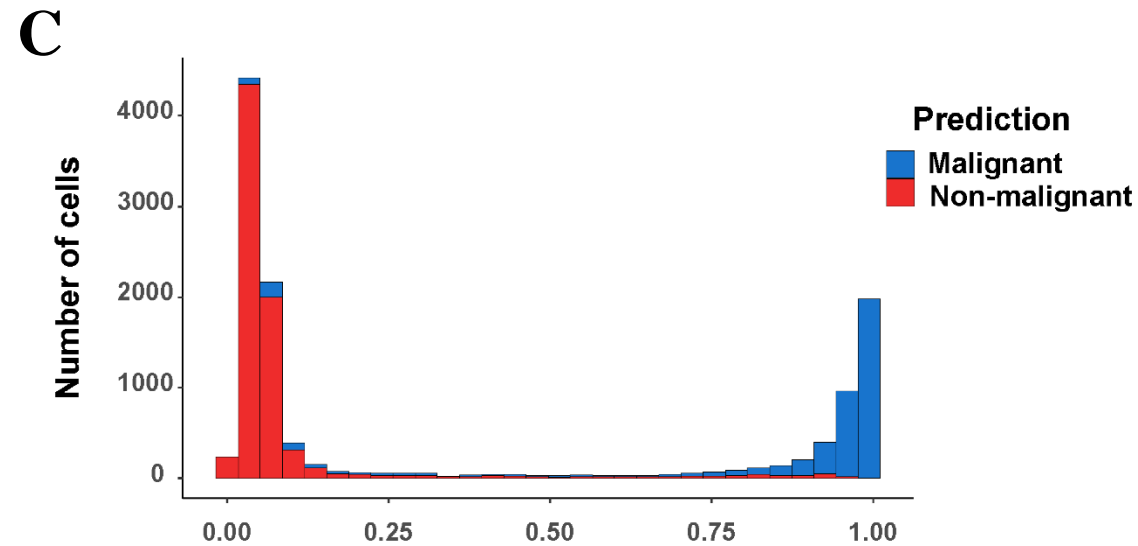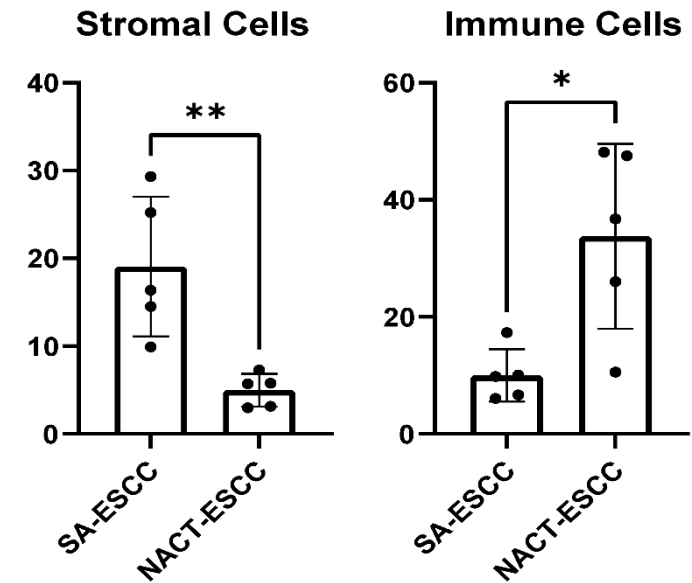

Supplement: Supplementary file 4 — Supplementary Figure 1 [file 41389_2021_359_MOESM4_ESM.pdf]

# A

## Normal

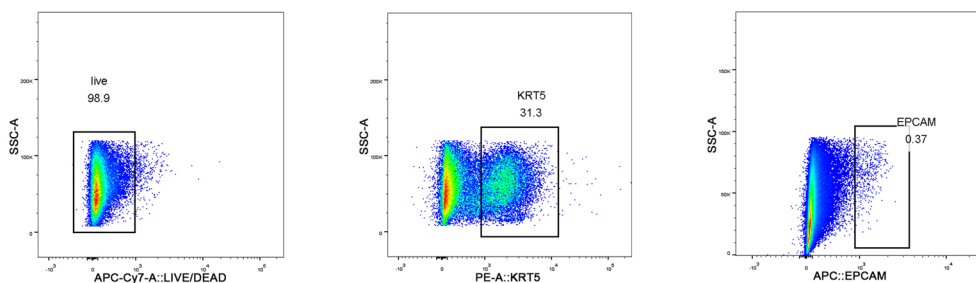

## SA-ESCC

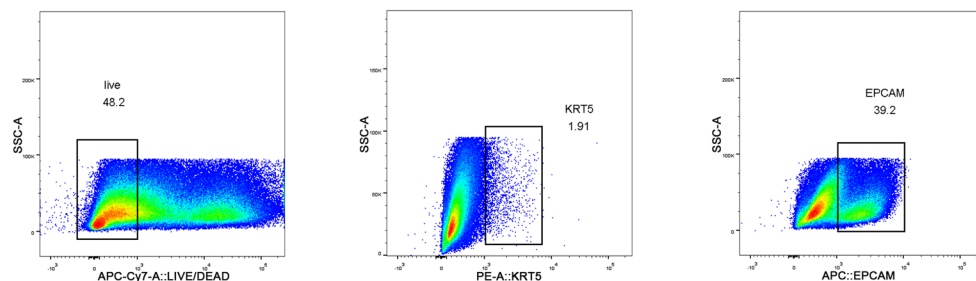

## NACT-ESCC

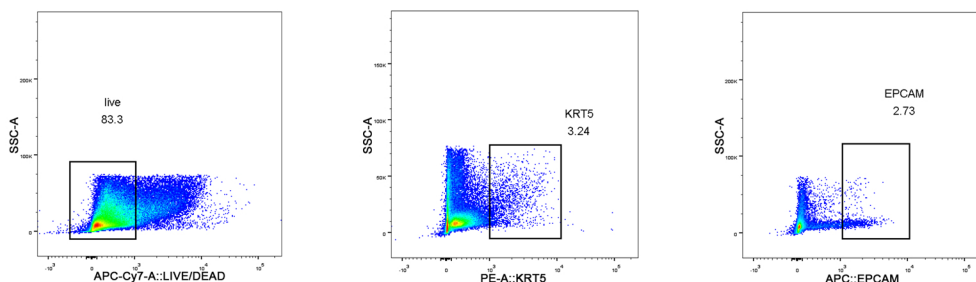

# B

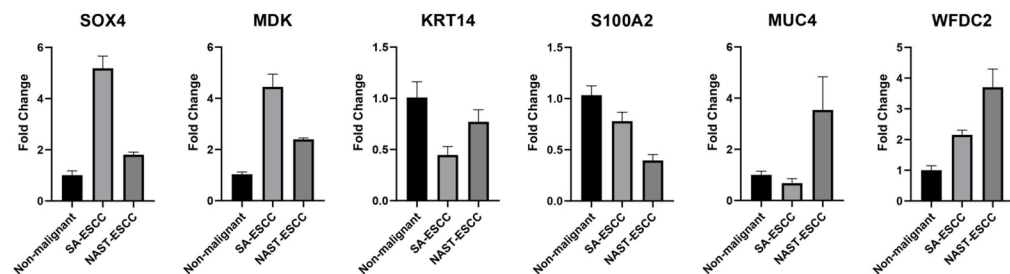

Supplement: Supplementary file 5 — Supplementary Figure 2 [file 41389_2021_359_MOESM5_ESM.pdf]

# A

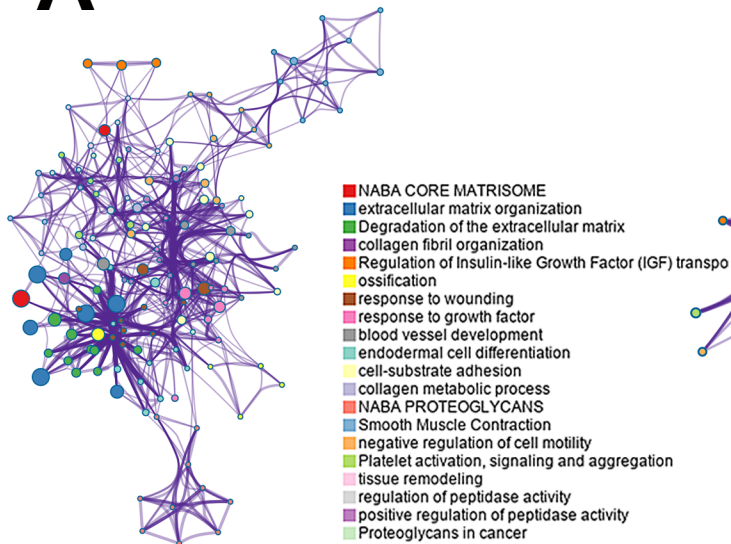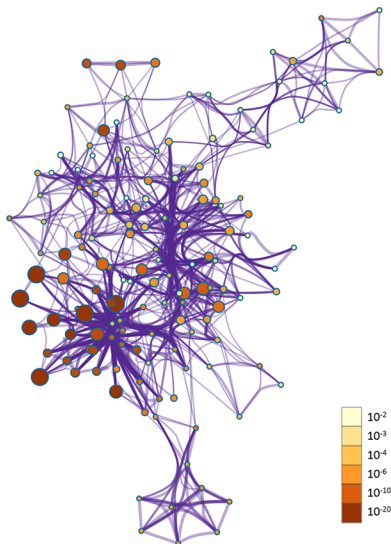

# B

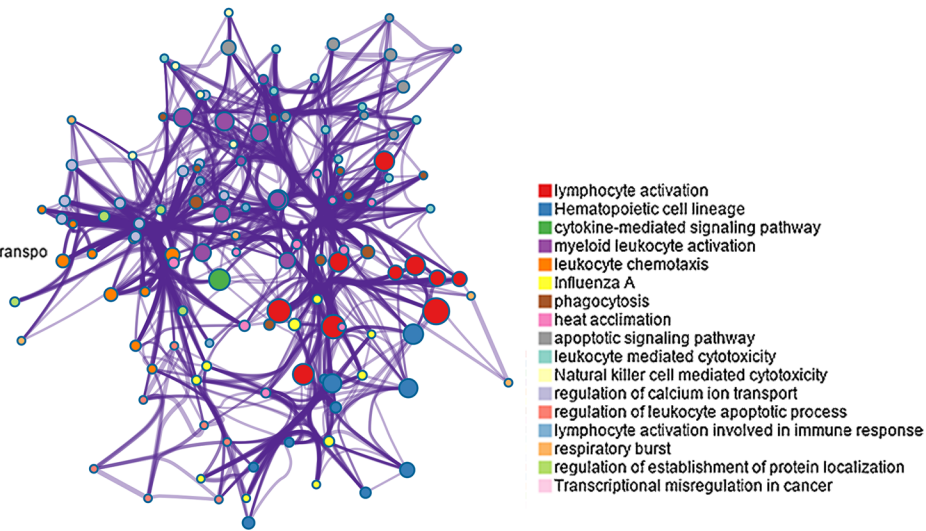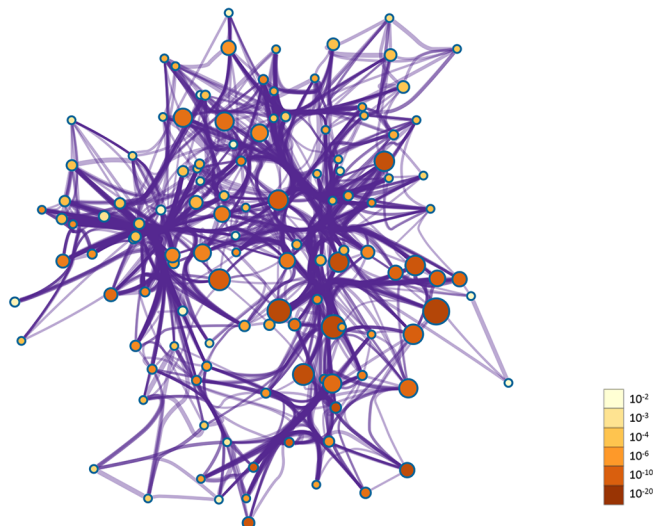

Supplement: Supplementary file 6 — Supplementary Figure 3 [file 41389_2021_359_MOESM6_ESM.pdf]

A

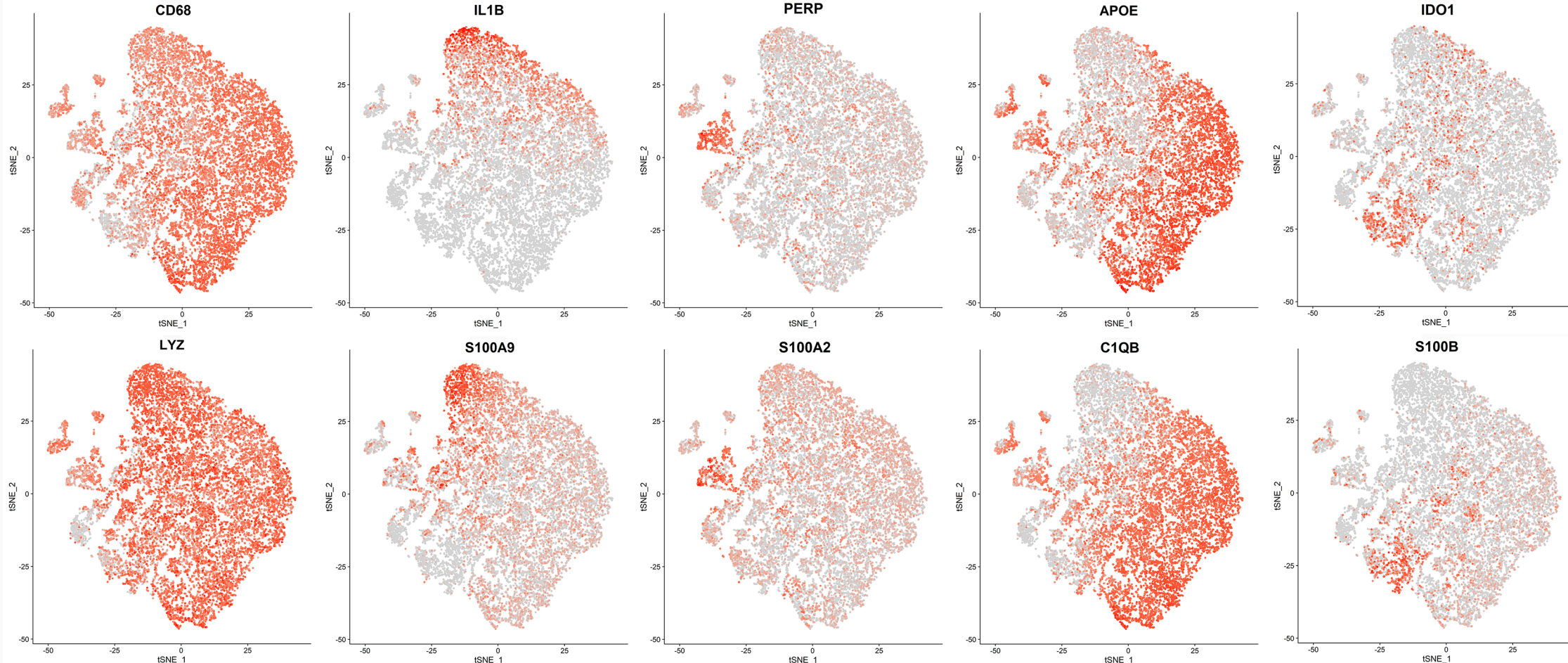

B

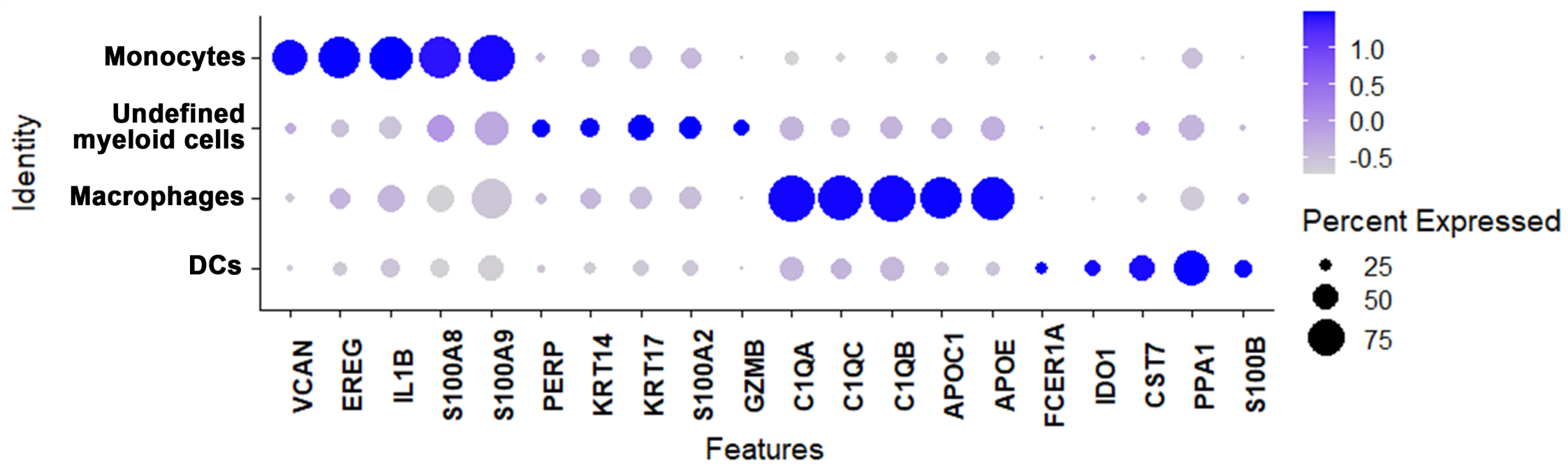

C

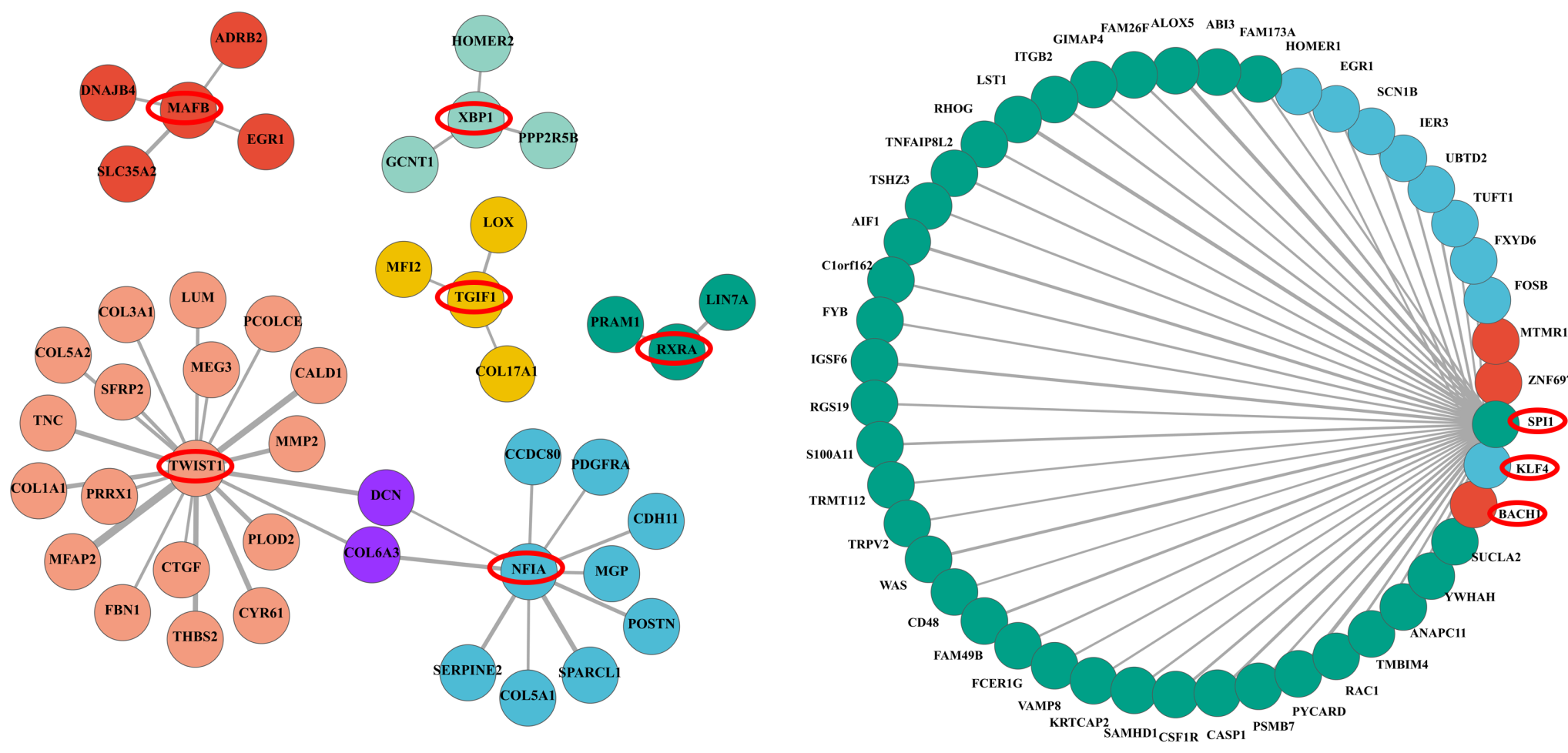

Supplement: Supplementary file 8 — Supplementary Figure 5 [file 41389_2021_359_MOESM8_ESM.pdf]

**A**

DAPI

APOE

SPP1

NACT-ESCC

DAPI

APOE

SPP1

SA-ESCC

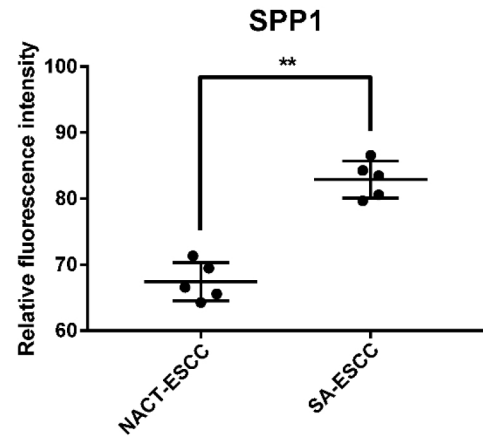**B**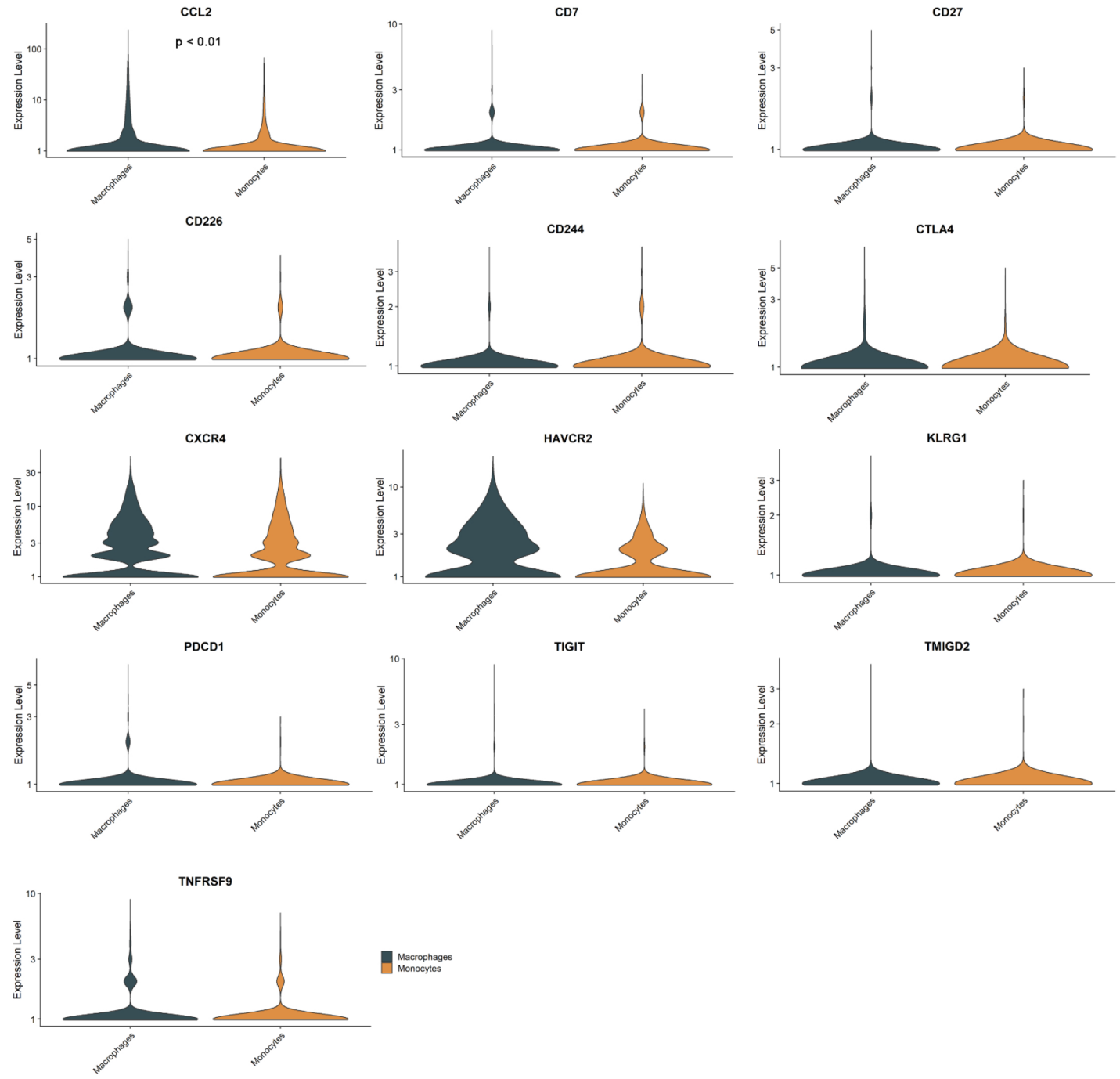

Supplement: Supplementary file 9 — Supplementary Figure 6 [file 41389_2021_359_MOESM9_ESM.pdf]

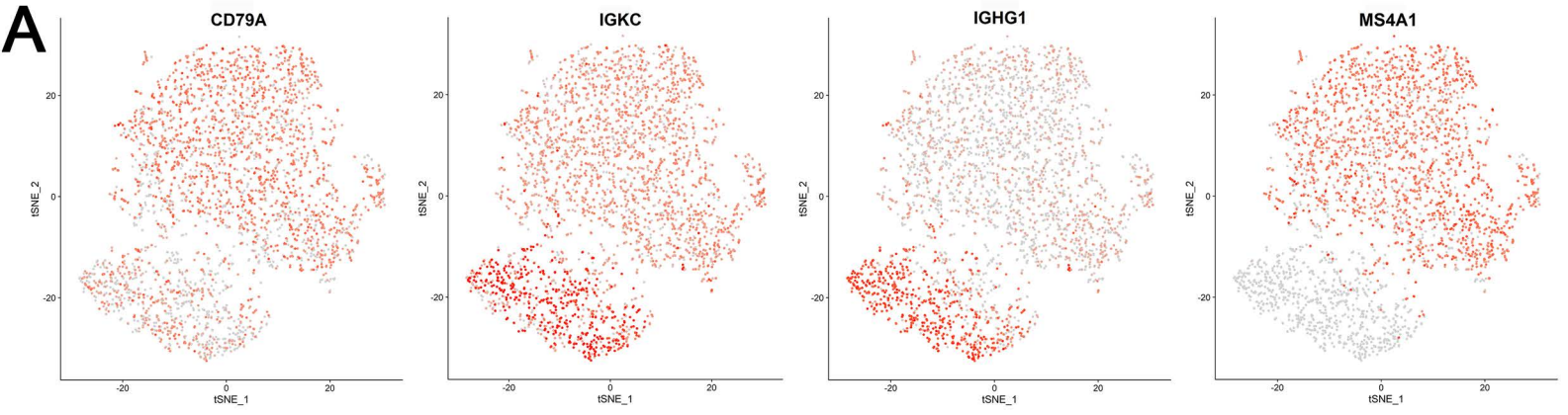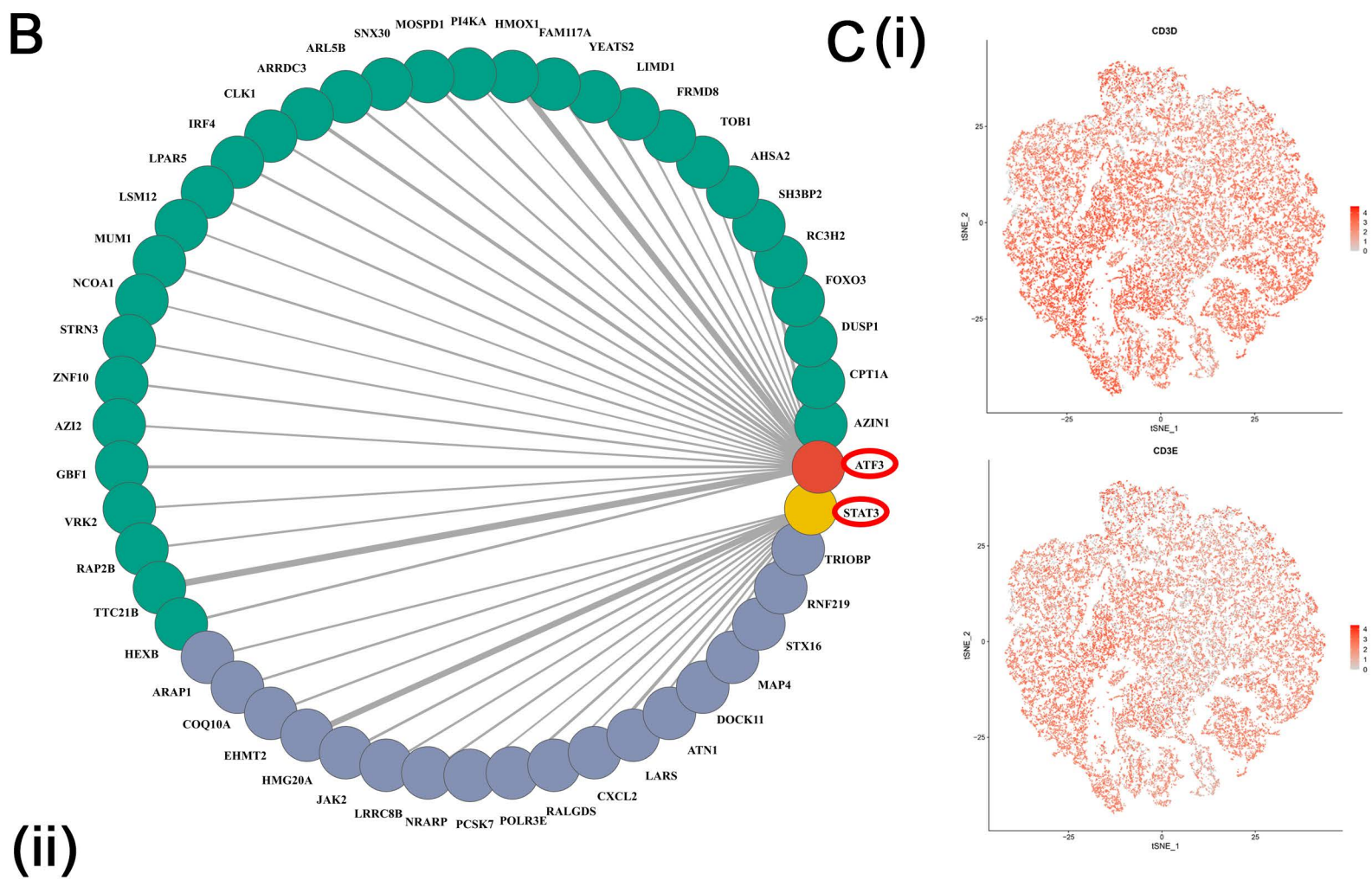

Supplement: Supplementary file 10 — Supplementary Figure 7 [file 41389_2021_359_MOESM10_ESM.pdf]

**A**

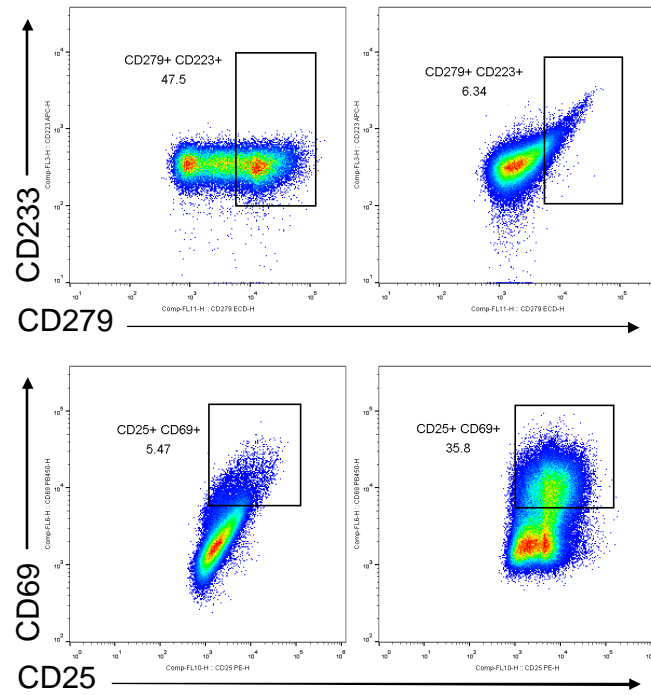

CD25+ CD69+ Activated T cell

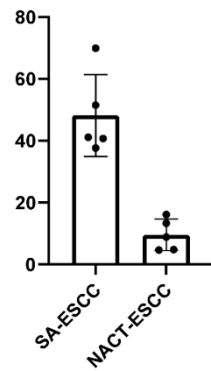

CD279+ CD223+ Exhausted T cell

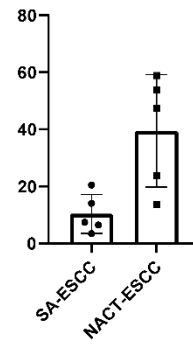

**B**

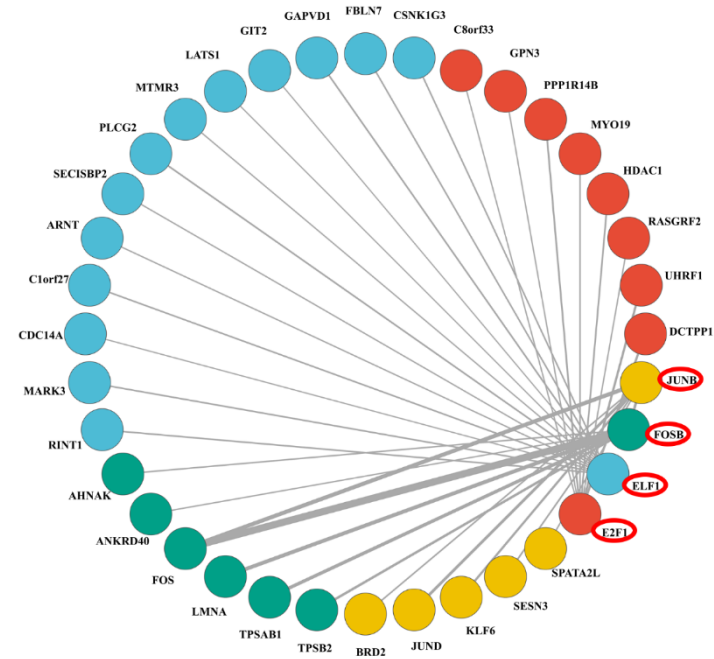

**C**

**LAG3**

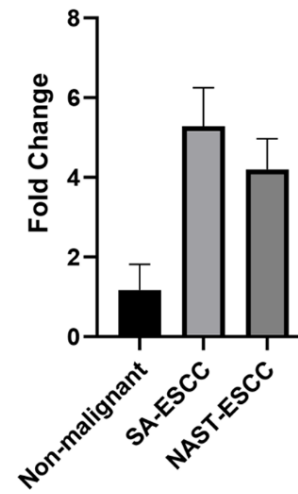

**HAVCR2**

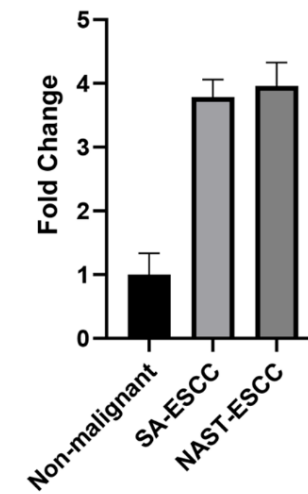

Supplement: Supplementary file 11 — Supplementary Figure 8 [file 41389_2021_359_MOESM11_ESM.pdf]

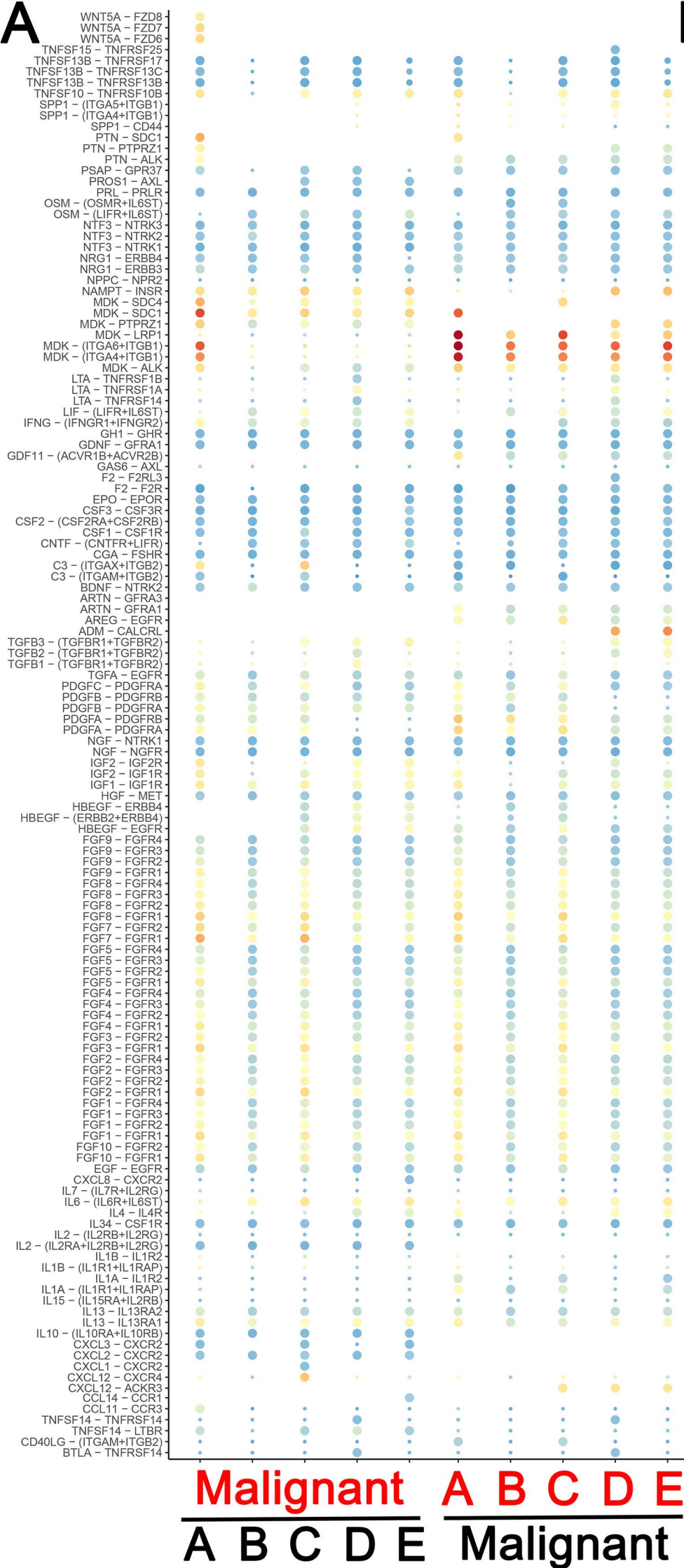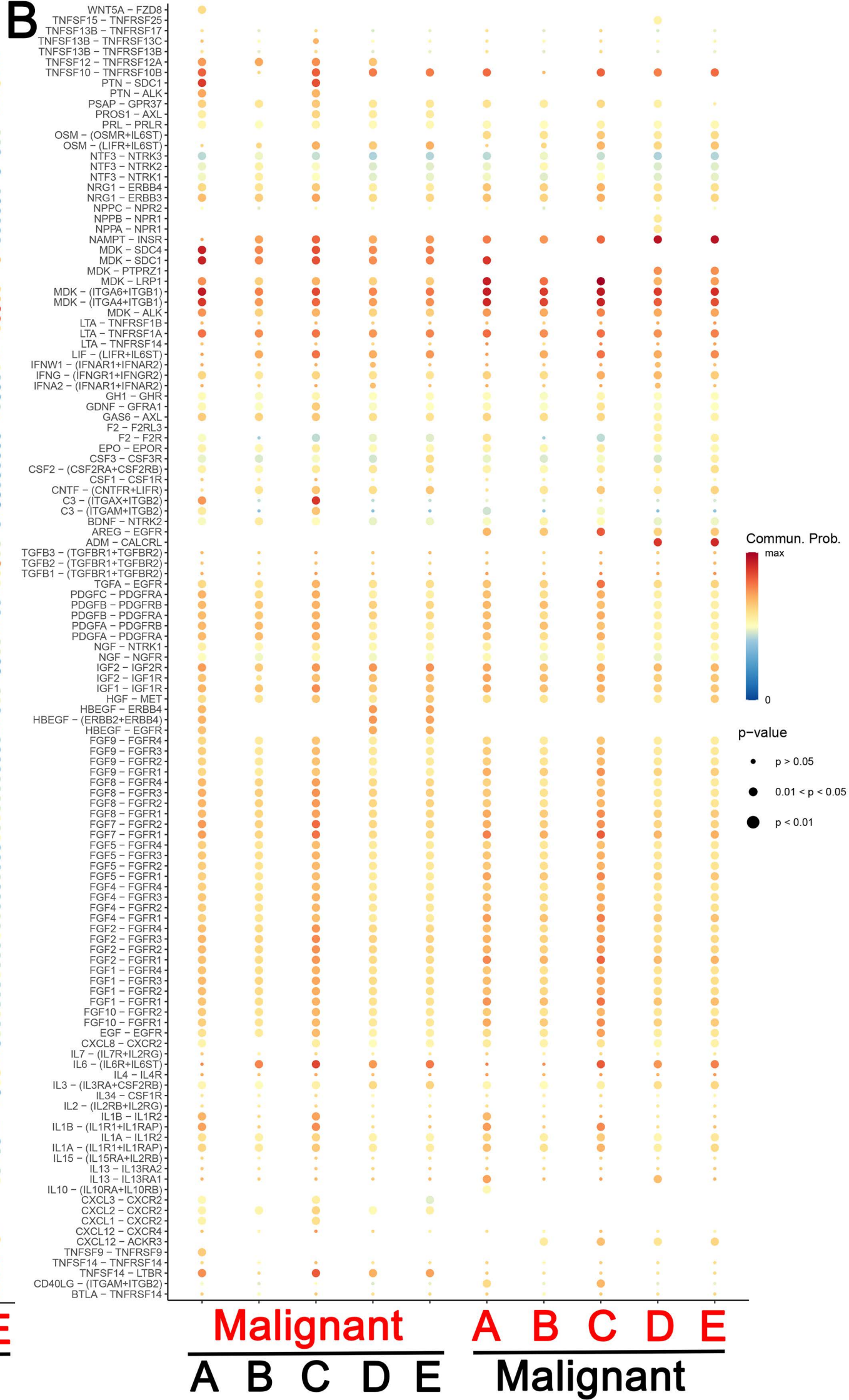

Supplement: Supplementary file 13 — Supplementary Figure 10 [file 41389_2021_359_MOESM13_ESM.pdf]

A

Dim 2

Dim 1

B

Pathway distance

Group

● NACT-ESCC

□ SA-ESCC

Cluster

● 1

● 2

● 3

● 4

Commun.Prob.

● 0.1

● 0.5

● 0.9

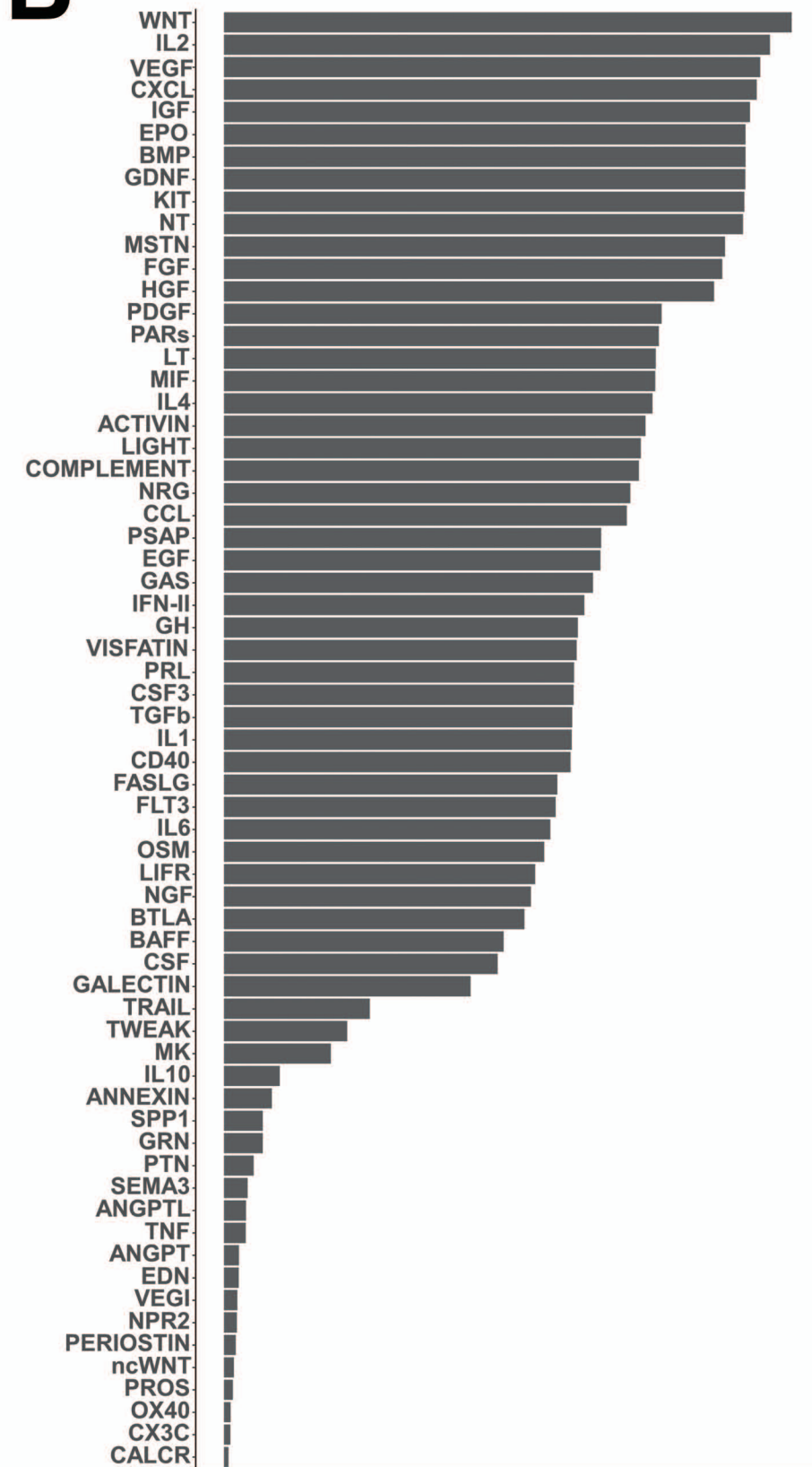

Supplement: Supplementary file 15 — Supplementary Figure 12 [file 41389_2021_359_MOESM15_ESM.pdf]
