# Supplementary material for: Dissecting the single-cell transcriptome network in patients with esophageal squamous cell carcinoma receiving operative paclitaxel plus platinum chemotherapy
Source: Oncogenesis. 2021 Oct 26;10(10):71. doi: 10.1038/s41389-021-00359-2 (PMC8546051; doi:10.1038/s41389-021-00359-2)
Supplement: Supplementary file 7 — Supplementary Figure 4 [file 41389_2021_359_MOESM7_ESM.pdf]

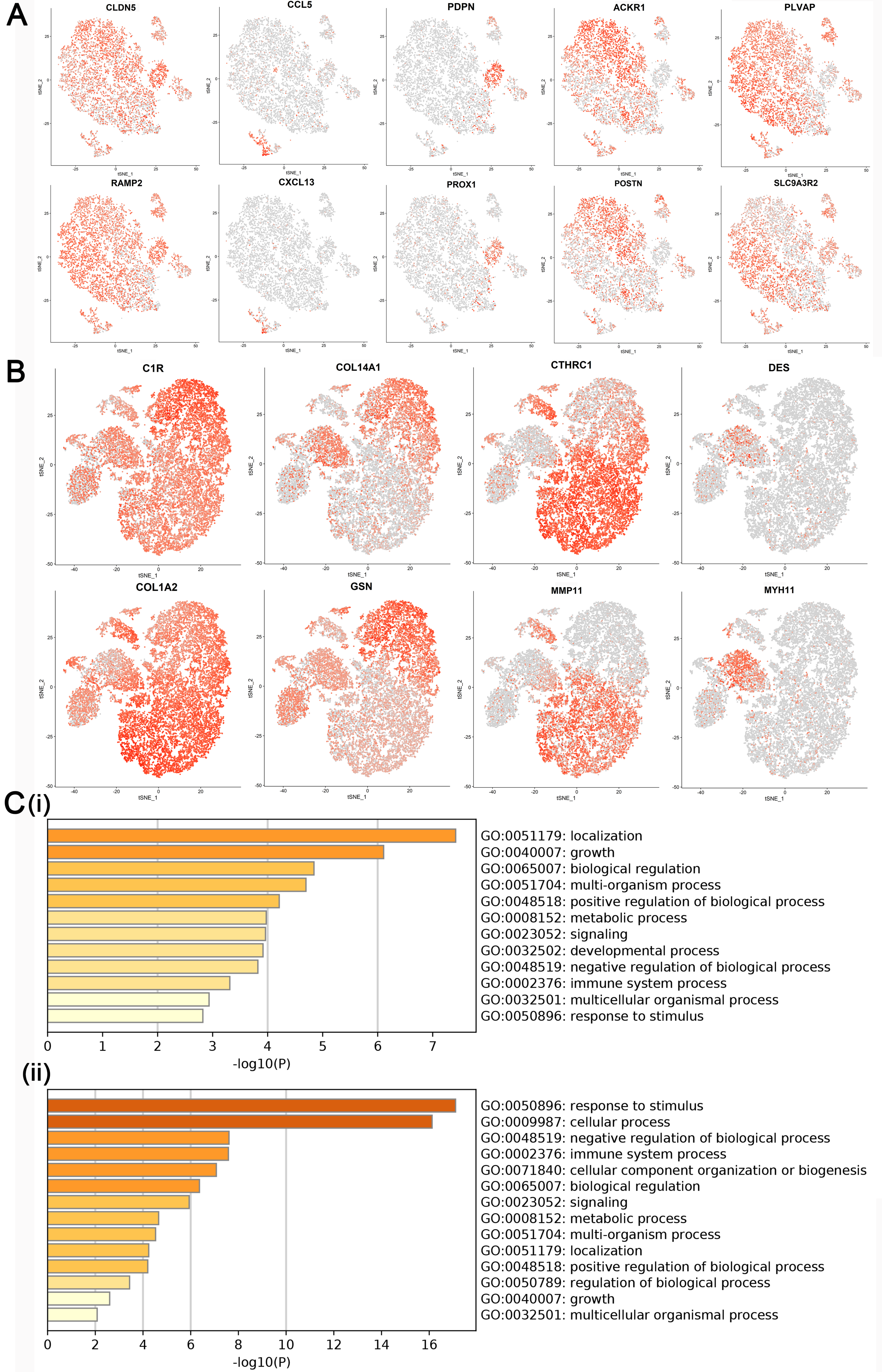
A
CLDN5
CCL5
PDPN
ACKR1
PLVAP
RAMP2
CXCL13
PROX1
POSTN
SLC9A3R2
tSNE_1
tSNE_2
B
C1R
COL14A1
CTHRC1
DES
COL1A2
GSN
MMP11
MYH11
C(i)
GO:0051179: localization
GO:0040007: growth
GO:0065007: biological regulation
GO:0051704: multi-organism process
GO:0048518: positive regulation of biological process
GO:0008152: metabolic process
GO:0023052: signaling
GO:0032502: developmental process
GO:0048519: negative regulation of biological process
GO:0002376: immune system process
GO:0032501: multicellular organismal process
GO:0050896: response to stimulus
-log10(P)
(ii)
GO:0050896: response to stimulus
GO:0009987: cellular process
GO:0048519: negative regulation of biological process
GO:0002376: immune system process
GO:0071840: cellular component organization or biogenesis
GO:0065007: biological regulation
GO:0023052: signaling
GO:0008152: metabolic process
GO:0051704: multi-organism process
GO:0051179: localization
GO:0048518: positive regulation of biological process
GO:0050789: regulation of biological process
GO:0040007: growth
GO:0032501: multicellular organismal process
-log10(P)
