# Supplementary material for: Dissecting the single-cell transcriptome network in patients with esophageal squamous cell carcinoma receiving operative paclitaxel plus platinum chemotherapy
Source: Oncogenesis. 2021 Oct 26;10(10):71. doi: 10.1038/s41389-021-00359-2 (PMC8546051; doi:10.1038/s41389-021-00359-2)
Supplement: Supplementary file 14 — Supplementary Figure 11 [file 41389_2021_359_MOESM14_ESM.pdf]

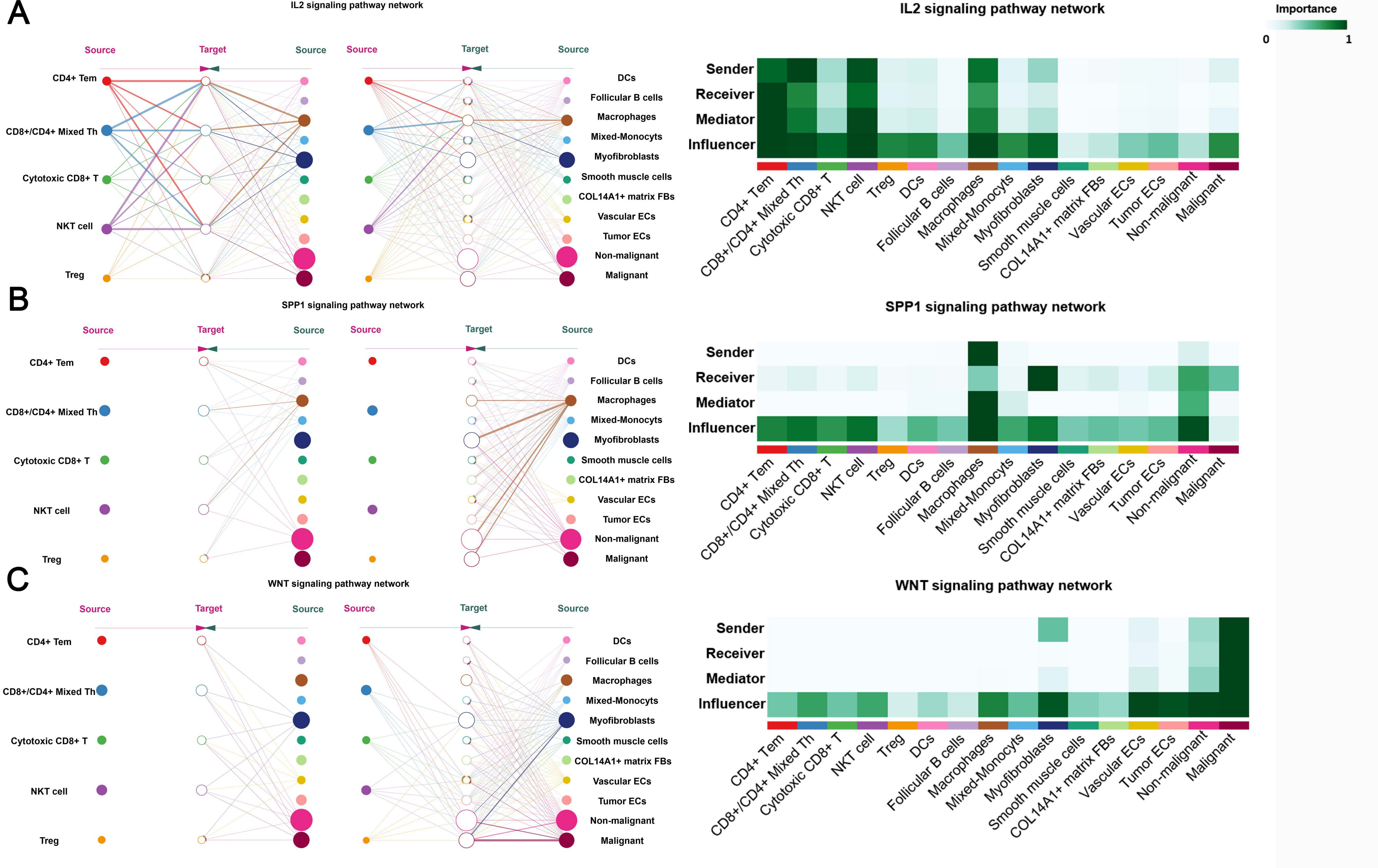
A
IL2 signaling pathway network
Source
Target
Source
CD4+ Tem
CD8+/CD4+ Mixed Th
Cytotoxic CD8+ T
NKT cell
Treg
DCs
Follicular B cells
Macrophages
Mixed-Monocyts
Myofibroblasts
Smooth muscle cells
COL14A1+ matrix FBs
Vascular ECs
Tumor ECs
Non-malignant
Malignant
Importance
0
1
Sender
Receiver
Mediator
Influencer
B
SPP1 signaling pathway network
C
WNT signaling pathway network
