# Supplementary material for: Dissecting the single-cell transcriptome network in patients with esophageal squamous cell carcinoma receiving operative paclitaxel plus platinum chemotherapy
Source: Oncogenesis. 2021 Oct 26;10(10):71. doi: 10.1038/s41389-021-00359-2 (PMC8546051; doi:10.1038/s41389-021-00359-2)
Supplement: Supplementary file 12 — Supplementary Figure 9 [file 41389_2021_359_MOESM12_ESM.pdf]

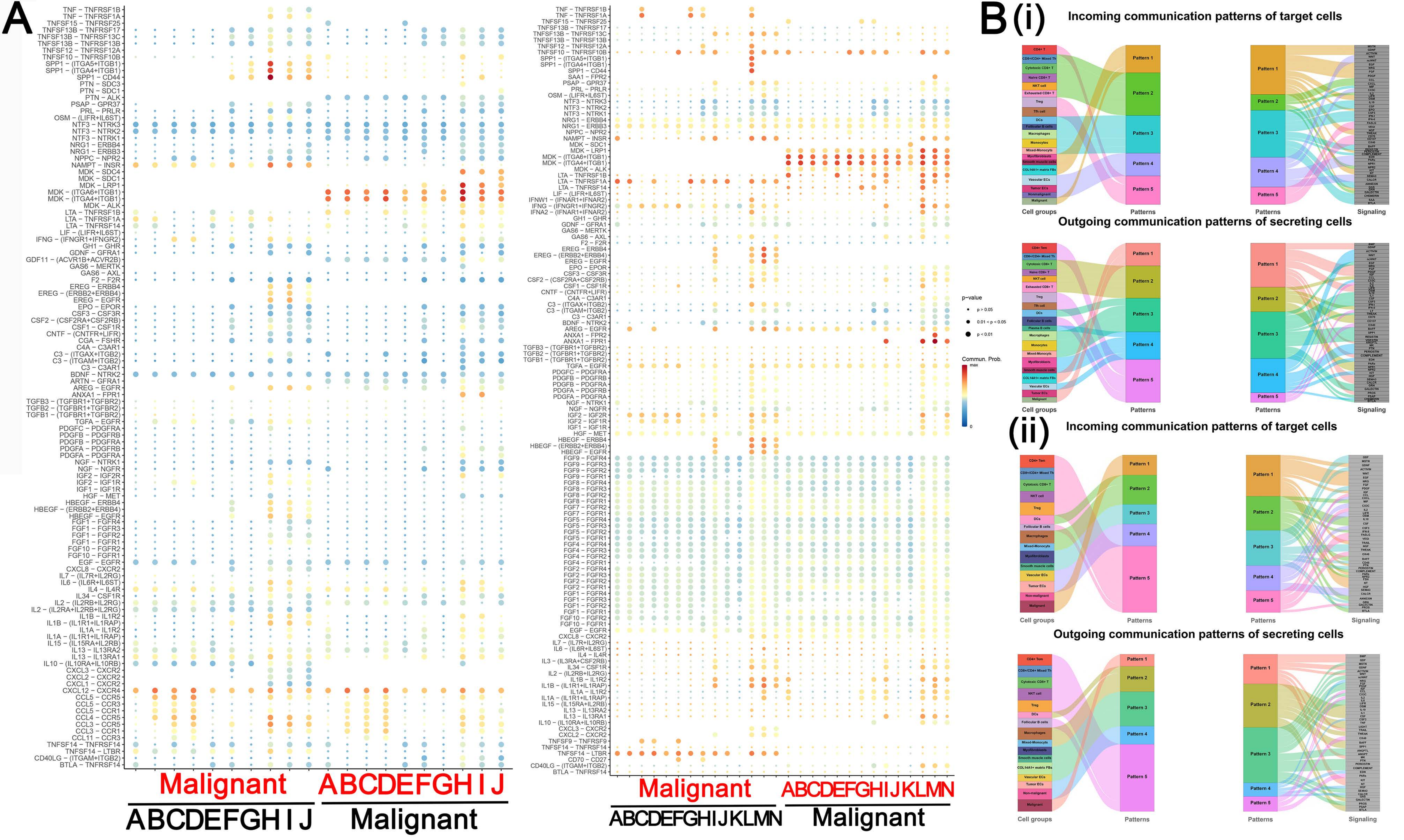
A
Malignant
ABCDEFGHIJ
ABCDEFGHIJ
Malignant
Malignant
ABCDEFGHIJKLMN
ABCDEFGHIJKLMN
Malignant
p-value
p > 0.05
0.01 < p < 0.05
p < 0.01
Commun. Prob.
max
0
B(i)
Incoming communication patterns of target cells
Outgoing communication patterns of secreting cells
(ii)
Incoming communication patterns of target cells
Outgoing communication patterns of secreting cells
Cell groups
Patterns
Signaling
Pattern 1
Pattern 2
Pattern 3
Pattern 4
Pattern 5
